# Comparative Evaluation of Plant-Derived Virus-like Particles as Intratumoral Immunotherapy Agents

**DOI:** 10.3390/vaccines14080697

**Published:** 2026-08-12

**Authors:** Anete Ogrina-Komarova, Zane Kalnina, Rebeka Racina, Vilija Zeltina, Ramona Petrovska, Ina Balke, Patricija Zaremba, Krista Resne, Juris Jansons, Andris Zeltins

**Affiliations:** 1 National Institute of Research and Innovation (NIRI), LV-1006 Riga, Latviajuris.jansons@niri.lv (J.J.); 2 Faculty of Medicine, Riga Stradins University, Dzirciema 16, LV-1007 Riga, Latvia

**Keywords:** plant-derived virus-like particles, intratumoral immunotherapy, melanoma, in situ vaccination

## Abstract

**Background:** Plant-derived virus-like particles (VLPs) are emerging nanoplatforms for local cancer immunotherapy, yet their relative performance across structurally distinct particles remains insufficiently defined. **Methods:** We performed a comparative benchmarking study of eleven plant-derived VLPs spanning diverse architectures and functional properties using an integrated workflow of physicochemical characterization, immune-functional profiling, and in vivo evaluation. All VLPs were produced in endotoxin-minimized ClearColi BL21 (DE3), enabling assessment of intrinsic particle-associated immunostimulatory activity with reduced bacterial endotoxin confounding. **Results:** In vitro, several VLPs stimulated macrophage-associated responses and enhanced tumor cell killing, although classical M1/M2 polarization markers in RAW264.7 cells did not consistently predict functional cytotoxicity. In a subset of candidates, HEK-TLR3 reporter activity varied substantially under RNA-normalized conditions and was not predicted solely by total RNA content or apparent RNA size distribution. Five candidates were advanced to intratumoral evaluation in the male-derived B16-F10 melanoma model, where CCMV-ss and CMVtt showed trends toward reduced tumor progression and increased immune cell infiltration in male mice. Furthermore, host sex-associated differences in baseline immune features were observed, though these must be interpreted with caution given the H-Y antigen-driven immunogenicity inherent to the male-derived B16-F10 model in female hosts. **Conclusions:** This study establishes a standardized comparative framework linking plant VLP properties with immune-functional performance and identifies CCMV-ss and CMVtt as promising candidates for further development as locally administered cancer immunotherapy nanoplatforms.

## 1. Introduction

Cancer immunotherapy seeks to overcome local immune suppression within the tumor microenvironment (TME) and restore effective antitumor immunity [1]. In this context, intratumoral delivery has emerged as an attractive strategy to concentrate immunostimulatory activity at the tumor site while promoting local inflammatory remodeling and endogenous antigen release [2]. A continuing challenge, however, is the identification of safe, scalable, and effective particulate platforms capable of inducing robust local immune activation without confounding effects from manufacturing-derived impurities [1,2,3].

Plant-derived VLPs are promising candidates for locally administered cancer immunotherapy. These particles do not directly lyse tumor cells; rather, when delivered into tumors, they can act as immunostimulatory scaffolds that engage innate immune pathways and reshape the TME. Previous studies with plant viral nanoparticles have shown induction of inflammatory cytokines and recruitment or activation of innate immune populations, changes that may facilitate tumor cell clearance, release of endogenous tumor antigens, and subsequent development of adaptive antitumor responses [4,5]. In this setting, plant VLPs may act as local immunostimulatory scaffolds that amplify endogenous antitumor immunity without requiring prior definition of tumor-specific target antigens. More broadly, VLPs are well known to be highly immunogenic particulate platforms, and previous studies, including our own, have shown that plant VLPs can elicit strong immune responses, including humoral immunity [6,7,8,9,10,11].

The biological activity of plant VLPs is likely influenced by multiple particle-associated properties, including capsid architecture, morphology, multivalent surface organization, and associated nucleic acid cargo [12,13,14,15]. Their repetitive nanoscale structure can enhance immune recognition, while encapsidated host-derived or recombinant RNAs may contribute to activation of innate sensing pathways. In myeloid cells, pattern recognition receptors (PRRs), including endosomal Toll-like receptors such as TLR3 and TLR7, can respond to nucleic acid-associated signals derived from viral particles or VLP preparations [16,17]. Despite this potential, how structural and cargo-associated features collectively influence immunostimulatory performance remains incompletely defined across different plant VLP platforms.

Several plant viral nanoparticles have shown antitumor activity in preclinical models, including strong efficacy of cowpea mosaic virus (CPMV) in B16-F10 melanoma and ID8-Defb29/Vegf-A ovarian cancer models, as well as therapeutic activity of potato virus X (PVX) in lymphoma models [4,16,17,18]. In contrast, other plant virus-derived particles have shown limited activity in melanoma or ovarian tumor settings [19]. These findings suggest that plant VLPs represent a promising but heterogeneous class of immunotherapeutic nanoplatforms, for which performance may depend strongly on particle-specific properties. However, most published studies have evaluated individual candidates under non-uniform experimental conditions, making it difficult to compare relative activity across platforms or to distinguish intrinsic particle effects from preparation-related variables such as bacterial endotoxin contamination.

This limitation is particularly important for translation, because candidate prioritization requires side-by-side evaluation of structurally diverse particles produced and tested under standardized conditions. Comparative studies that include both icosahedral and filamentous plant VLPs, while also minimizing endotoxin-related confounding, remain limited. In addition, host sex and tumor model context can influence antitumor immunity and therapeutic outcomes in transplantable tumor models, particularly when the biological sex origin of the tumor cell line introduces an additional experimental variable [20]. These considerations underscore the need for a standardized comparative framework linking plant VLP platform properties with biological performance after local administration.

Here, we systematically evaluated a deliberately heterogeneous panel of eleven plant-derived VLPs as candidates for intratumoral cancer immunotherapy. Rather than restricting the study to only apparently optimal particles, we included VLPs spanning diverse structural and functional properties to enable benchmarking across a broader performance range. The panel comprised plant virus coat protein assemblies derived from eggplant mosaic virus (EMV), scrophularia mottle virus (SrMV), broad bean mottle virus (BBMV), apple mosaic virus (ApMV), potato virus Y (PVY), potato virus M (PVM), potato virus X (PVX), cocksfoot mottle virus (CfMV), and rice yellow mottle virus (RYMV), together with engineered analogues including the salt-stable cowpea chlorotic mottle virus variant (CCMV-ss) [21] and tetanus toxoid epitope-containing cucumber mosaic virus (CMVtt) [7]. Recombinant production was performed in *Escherichia coli*, with BBMV, SrMV, and PVX VLPs produced in this system for the first time. In addition, all VLPs except CCMV-ss and CMVtt are reported here for the first time as produced in the endotoxin-minimized ClearColi^TM^ strain, enabling subsequent cell-based and in vivo studies with reduced endotoxin-related confounding.

To compare these platforms systematically, we implemented a standardized side-by-side evaluation workflow integrating in vitro and in vivo analyses. Initial screening assessed cytocompatibility across a range of VLP concentrations in RAW264.7 macrophages. Selected candidates were then evaluated for macrophage-associated responses through analysis of surface marker expression and cytokine production. Functional antitumor activity was examined in macrophage–tumor co-culture assays using B16-F10-luc2 melanoma cells at varying effector-to-target ratios following stimulation with five selected VLPs. To further explore innate immune activation pathways, HEK-TLR3 reporter assays were performed on a subset of VLPs to assess RNA-associated signaling. Finally, five VLP candidates were advanced to intratumoral evaluation in the male-derived B16-F10 melanoma model in male and female C57BL/6 mice, with untreated and PBS-treated groups included as controls. Overall, this study was designed to establish a comparative framework for prioritizing plant VLP candidates and identifying particle-associated features linked to immunostimulatory and antitumor activity, rather than to provide a definitive mechanistic dissection of all pathways involved.

## 2. Materials and Methods

### 2.1. Expression and Purification of Plant Virus Coat Proteins (CPs) in Endotoxin-Minimized ClearColi BL21 (DE3) Cells

Plant virus coat protein (CP) expression vectors encoding eleven plant virus CPs were transformed into endotoxin-minimized *E. coli* ClearColi BL21 (DE3) cells (LGC, Guildford, UK). The constructs included pET42-EMV [10], pET28-PVM [6], pET28-PVY [22], pET28-RYMV, pET28-CfMV [7,23], pET42-CCMV-ss [8], and pET28-CMVtt [7]. Two constructs were generated from viruses isolated from host plants: ApMV from infected apple leaves and PVX from infected potato plantlets (GenBank sequence GQ496608.1). Their coat protein (CP) genes were amplified and subsequently cloned into the pET28 expression vector (sequence for ApMV CP provided in Supplementary Figure S1). CP genes from two additional plant viruses were obtained by gene synthesis: pET42-SrMV and pET42-BBMV, based on sequences from the GenBank database (NC_011537.1 for SrMV and NC_004006 for BBMV). For plant CP genes containing rare AA codons, the pET28-CfMV and pET28-ApMV vectors were co-transformed with the pACYC-RIL plasmid (Agilent Technologies, Santa Clara, CA, USA). The best-expressing clones for each construct were selected and cultivated in 2 × TY medium (1.6% tryptone, 1.0% yeast extract, 0.5% NaCl) supplemented with the appropriate antibiotic (kanamycin, 25 µg/mL, for pET42 and pET28-derived constructs; chloramphenicol, 25 µg/mL, for pACYC derivatives). *E. coli* cultures were grown in shaking flasks using an Ecotron HT tabletop incubator (Infors, Bottmingen, Switzerland) at 30 °C and 200 rpm until the optical density at 600 nm (OD_600_) reached 0.8–1.0. Protein expression was induced with 0.2 mM isopropyl β-D-1-thiogalactopyranoside (IPTG), and supplemented with 5 mM MgCl_2_, 2 mM CaCl_2_ for CfMV, RYMV, CCMV-ss, BBMV and the cultures were further incubated at 20 °C for 18 h. Cells were harvested by low-speed centrifugation (2600× *g*, 5 min, 5 °C; Eppendorf 5804R, Hamburg, Germany) and stored at −70 °C until further use.

Cells containing EMV, PVY, PVX and ApMV CPs were thawed on ice, resuspended in 1× PBS, and disrupted using an ultrasonic homogenizer (Hielscher UP200, Teltow, Germany, 70% power, 50% pulse, 16 min) on ice. The same procedure was applied to the remaining CPs using the following buffers: RYMV, CfMV, and BBMV in 15 mM KH_2_PO_4_, pH 5.5; PVM in 20 mM Tris, pH 8.0; CCMV-ss in 15 mM Na_2_HPO_4_ pH 7.5, 150 mM NaCl; and CMVtt in 50 mM sodium citrate, 5 mM sodium borate, 5 mM EDTA, pH 9.0. CCMV-ss buffer additionally contained 0.5 M urea and 1 mM PMSF; RYMV and CfMV buffers contained 1 mM PMSF; and ApMV buffers contained 1 mM CaCl_2_. Lysates were clarified by centrifugation at 15,000× *g* for 10 min at 5 °C to remove cellular debris, and supernatants were collected for subsequent purification.

Purification of all CPs was performed by ultracentrifugation (Optima L-100 XP, Beckman Coulter, Brea, CA, USA; Ti SW 32 rotor; 110,000× *g*, 6 h, 18 °C) in a 20–60% sucrose gradient prepared in the corresponding CP buffer supplemented with 0.5% Triton X-100, as described previously [8,9]. After ultracentrifugation, gradient fractions were collected and analysed by 12.5% SDS-PAGE to identify those containing the respective VLPs.

VLP-containing fractions were pooled, transferred to new ultracentrifuge tubes, and pelleted by ultracentrifugation (Optima L-100 XP, Beckman Coulter, USA; 70 Ti rotor; 50,000 rpm (250,000× *g*), 4 h, 4 °C). The resulting pellets were gently resuspended in the corresponding buffers. VLPs were further purified by ultracentrifugation through a 30% sucrose cushion prepared in the corresponding buffers supplemented with 0.5% Triton X-100 (Optima L-100 XP; 70 Ti rotor; 50,000 rpm, 4 h, 4 °C), and this step was repeated without Triton X-100 when necessary. Pellets were finally resuspended in the appropriate buffers and, if required, concentrated using Amicon Ultra-15 100 kDa centrifugal filter units (Merck Millipore, Burlington, MA, USA), and purified VLPs were stored at 4 °C.

Protein concentrations were determined using a Qubit 2.0 fluorometer (Thermo Fisher Scientific, Waltham, MA, USA) according to the Qubit Protein assay manufacturer’s instructions (Thermo Fisher Scientific, USA, cat. no. Q33211). All purification steps were monitored by 12.5% SDS-PAGE and agarose gel electrophoresis. Purified VLPs were stored at 4 °C and tested for endotoxin content using the Limulus amebocyte lysate (LAL) assay (Pierce LAL Chromogenic Endotoxin Quantitation Kit, Thermo Fisher Scientific, cat. no. 88282), showing <100 EU/mg protein [11].

### 2.2. Transmission Electron Microscopy (TEM)

Purified VLPs (1 mg/mL) were adsorbed onto carbon/formvar-coated 300-mesh copper grids (Agar Scientific, Rotherham, UK) for 3 min, washed with EDTA, and negatively stained with 0.5% aqueous uranyl acetate. Grids were examined using a JEM-1230 transmission electron microscope (JEOL, Tokyo, Japan) at an accelerating voltage of 100 kV, with at least five images captured per sample.

### 2.3. Dynamic Light Scattering (DLS)

Purified VLPs (1 mg/mL) were analyzed undiluted in a high-precision micro quartz cuvette (ZEN2112, Hellma Analytics, Müllheim, Germany; 12 µL sample volume) using a Zetasizer Nano ZS instrument (Malvern Instruments Ltd., Malvern, UK). Hydrodynamic diameters were determined from three consecutive measurements and analyzed using Zetasizer software (version 8.01). All DLS data and analysis outputs are provided in the Supplementary Information (Supplementary Figures S2–S5).

### 2.4. RNA Isolation and Quantification with TRI Reagent or NucleoSpin Virus Kit

To quantify RNA content in VLPs, 1 mL TRI Reagent was added to 200 µL of SrMV, PVM, RYMV, CCMV-ss, and CMVtt VLPs, vortexed for 5 s, and incubated for 5 min at RT. Chloroform (0.2 mL) was added, vortexed for 15 s, and incubated for 10 min at room temperature, followed by centrifugation (14,000 rpm, 4 °C, 15 min). The upper aqueous phase containing RNA was transferred to new tubes, mixed with 0.5 mL 2-propanol, incubated for 10 min at RT, and centrifuged (14,000 rpm, 10 min, 4 °C) to pellet RNA. Pellets were washed with 75% ethanol, briefly air-dried (5 min), resuspended in 100 µL DEPC-treated water, incubated at 55 °C for 10 min, and quantified by NanoDrop spectrophotometry (protein:RNA ratio ≈ 10:1). Isolated RNAs were analyzed by 1% agarose gel electrophoresis. RNA isolation was also performed using a column-based method according to the manufacturer’s instructions (NucleoSpin^®^ Virus Kit, Macherey-Nagel, Düren, Germany; cat. no. 740983.50).

### 2.5. HEK-Blue^TM^ TLR 3 Cell Assay

After RNA isolation, RNA yield was quantified via NanoDrop^TM^ 1000 (Thermo Fisher Scientific), and size distribution was evaluated by 1% agarose gel electrophoresis in 1× TBE with ethidium bromide, using RNA ladders (Thermo Fisher Scientific, #SM1821). Samples were prepared 1:1 with 2× RNA loading dye (Thermo Fisher Scientific), denatured at 70 °C for 10 min, and chilled on ice for 3 min prior to loading. Gels were visualized by ethidium bromide staining.

TLR3-stimulatory activity of RNA from SrMV, PVM, CCMV-ss, RYMV and CMVtt intact VLPs was assessed in HEK-Blue^TM^ hTLR3 cells (InvivoGen, San Diego, CA, USA, Cat. No. hkb-htlr3) per the manufacturer’s instructions. Cells (50,000/well) were stimulated in triplicate with VLP doses equivalent to 250 or 500 ng RNA content, or 20 ng poly(I:C) HMW (InvivoGen, Cat. No. tlrl-pic-5) as positive control. TLR3 responses were normalized to poly(I:C)-stimulated controls.

### 2.6. Cell Lines

B16-F10 (ATCC, Manassas, VA, USA, CRL-6475) and B16-F10-luc2 (ATCC, USA, CRL-6475-LUC2) cells were cultured in 75 cm^2^ adherent Falcon flasks (Corning, Corning, NY, USA, cat. no. 353136) using DMEM (Gibco, Thermo Fisher Scientific, cat. no. 31331093) supplemented with 10% FBS (Thermo Fisher Scientific, cat. no. 10270-106), 1% penicillin-streptomycin (Merck, Darmstadt, Germany, cat. no. P4333), and 2 mM L-glutamine at 37 °C in a humidified 5% CO_2_ atmosphere (Panasonic, Kadoma City, Japan, MCO-18AC-PE). B16-F10-luc2 cultures were additionally maintained with 10 µg/mL blasticidin (Thermo Fisher Scientific, cat. no. BP264725), which was omitted prior to experimental use.

Cells in their exponential growth phase were rinsed three times with 1× PBS, detached with 0.5% Trypsin-EDTA (Thermo Fisher Scientific, cat. no. 25300-054) for 7 min at 37 °C, neutralized with complete medium, pelleted by centrifugation (300× *g*, 5 min), washed three times with cold PBS, resuspended, and counted using a Cellometer Mini (Nexcelom Bioscience, Lawrence, MA, USA). Aliquots of 1 × 10^5^ cells were kept on ice water until injection. Mycoplasma contamination was ruled out routinely using the MycoAlert Mycoplasma Detection Kit (Lonza, Guangzhou, China, cat. no. LT07-318).

Murine RAW264.7 macrophages (ATCC, USA, cat. no. TIB-71) were maintained in DMEM (Gibco, Thermo Fisher Scientific, cat. no. 31331093) supplemented with 10% fetal bovine serum (FBS) (Sigma–Aldrich, St. Louis, MO, USA, cat. no. F7524), 1% penicillin-streptomycin (Euroclone, Pero, Italy, cat. no. ECB3001), and 2 mM L-glutamine (Sigma-Aldrich, USA, cat. no. C7513) at 37 °C in a humidified 5% CO_2_ atmosphere (Panasonic, Japan, MCO-18AC-PE). Cells were passaged at 60–75% confluency using cell scrapers and reseeded at a 1:3–1:5 dilution. Only cells at passages ≤ 25 were used for experiments to maintain phenotypic stability.

Cells were incubated in a humidified 5% CO_2_ incubator (Panasonic, Japan, MCO-18AC-PE) at 37 °C and passaged using 0.05% trypsin solution (Cat. No. 15400-054, Gibco, Life Technologies, Carlsbad, CA, USA).

### 2.7. Macrophage Viability Assay

RAW264.7 macrophages (10^5^ cells/well) were seeded in 96-well plates (Sarstedt, Hildesheim, Germany, cat. no. 83.3924) and allowed to attach overnight at 37 °C in 5% CO_2_. The following day, cells were exposed to eleven plant-derived VLPs at concentrations of 1, 2, and 5 µg/mL in complete DMEM supplemented with 10% FBS, 1% penicillin–streptomycin, and 2 mM L-glutamine. Two technical replicate wells were prepared for each experimental condition. Untreated controls received fresh medium only, whereas positive controls were treated with lipopolysaccharide (LPS; 100 ng/mL). After 20 h of incubation at 37 °C, cells were examined using a light microscope (Leica) at 20× and 40× magnification to assess morphological changes. Cells from the technical replicate wells were then pooled, stained with Live/Dead Aqua viability dye (Thermo Fisher Scientific, USA, cat. no. L34957) according to the manufacturer’s instructions, and analyzed by flow cytometry to quantify cell viability.

Flow cytometry data from this screening assay are presented descriptively to illustrate relative viability trends across the VLP panel rather than to provide quantitative statistical comparisons between treatment groups.

### 2.8. Macrophage M1/M2-Associated Activation Marker Assay

RAW264.7 macrophages (10^5^ cells/mL) were seeded in 96-well plates (Sarstedt, Germany, cat. no. 83.3924) and allowed to attach overnight at 37 °C in 5% CO_2_ in a humidified chamber (Panasonic, Japan, MCO-18AC-PE). The following day, cells were exposed to VLPs derived from 11 different plant viruses at 2 µg/mL diluted in complete DMEM medium (DMEM supplemented with 10% FBS, 1% penicillin–streptomycin, and 2 mM L-glutamine). Two technical replicate wells were prepared for each experimental condition. Untreated controls received fresh medium only, and M1 macrophage positive controls were treated with lipopolysaccharide (LPS; 100 ng/mL) and interferon-γ (IFN-γ; 20 ng/mL), while the M2 macrophage positive control was treated with interleukin-4 (IL-4; 20 ng/mL). After 20 h of incubation at 37 °C, morphology was assessed by light microscopy (Leica; 20× and 40× magnification; ≥3 images/well). Cells from the two technical replicate wells were harvested by gentle scraping and pooled to generate one sample per condition for flow cytometric analysis of M1/M2 phenotypic markers. The experiment was repeated in 24-well plates using 5 µg/mL SrMV, PVM, CCMV-ss, RYMV, or CMVtt VLPs, with M1-positive controls of 100 ng/mL LPS + 20 ng/mL IFN-γ, 100 ng/mL LPS + 100 ng/mL IFN-γ, and an M2-positive control of 20 ng/mL IL-4 for cell marker analysis. The LPS/IFN-γ and IL-4 conditions were included as reference stimuli for interpreting M1- and M2-associated marker patterns, rather than as definitive validation of stable macrophage polarization states.

Flow cytometry data from this screening assay are presented descriptively to illustrate relative macrophage activation trends across the VLP panel rather than to provide quantitative statistical comparisons between treatment groups.

FACS staining: Cells from the two technical replicate wells were pooled and stained with Live/Dead Aqua fixable viability dye (Invitrogen, Carlsbad, CA, USA, cat. no. L34957) for 30 min at 4 °C, washed, and split into two U-bottom 96-well plates.

Surface markers: Cells were stained for 30 min at 4 °C with FITC CD86 (Biolegend, San Diego, CA, USA, cat. No 105006), BV421 CD206 (BioLegend, cat. no. 141717).

Intracellular markers: Cells were fixed/permeabilized using BD Cytofix/Cytoperm Plus (cat. no. 554715), and stained for 30 min at 4 °C with APC-eFluor780 iNOS (Thermo Fisher Scientific, USA, cat. No. 47-5920-82) and APC Arginase 1 (Thermo Fisher Scientific, USA, cat. no. 17-3697-82).

Unstained controls were included to assess background fluorescence and support interpretation of positive staining populations. Representative unstained RAW264.7 macrophage control plots are provided in Supplementary Figure S6. Samples were washed with FACS buffer (PBS + 2% FBS), acquired on a Cytek Aurora spectral flow cytometer (Cytek Biosciences, Fremont, CA, USA), and analyzed using FlowJo v10.10.0 software (Tree Star, Ashland, OR, USA) and GraphPad Prism v8.4.2 software.

### 2.9. Co-Cultivation of Macrophages with B16-F10-luc2 Melanoma Cells for Bioluminescence Assay

B16-F10-luc2 melanoma cells were seeded in black 96-well plates (Corning, cat. no. CLS3603) at a density of 4 × 10^4^ cells/mL and allowed to adhere for 3 h at 37 °C in a humidified 5% CO_2_ atmosphere. RAW264.7 macrophages were then added to B16-F10-luc2 tumor cells at tumor-cell: macrophage ratios of 1:0.25, 1:0.5, 1:1, 1:2.5, and 1:3.5. Co-cultures were treated at the time of macrophage addition with plant-derived VLPs, 5 µg/mL; LPS, 100 ng/mL, plus IFN-γ, 100 ng/mL, as an M1-associated activation control; or IL-4, 20 ng/mL, as an M2-associated activation control. Additional controls included B16-F10-luc2 tumor cells treated with each corresponding stimulus alone, including VLPs, LPS/IFN-γ, or IL-4, as well as macrophage-only wells treated with each corresponding stimulus. Cells were incubated for 20 h at 37 °C in a humidified 5% CO_2_ atmosphere.

Following incubation, culture medium was aspirated and replaced with phenol red-free DMEM (Gibco, Thermo Fisher Scientific, USA, cat. no. 11039054) containing 150 µg/mL D-luciferin (Revvity, Waltham, MA, USA, cat. no. 122799A). After incubation for 10–15 min at 37 °C with gentle shaking, bioluminescence was measured using a Victor3V 1420-040 multilabel plate reader (PerkinElmer, Waltham, MA, USA). Luminescence was recorded as relative light units (RLU) per well.

The macrophage–tumor cell co-culture killing assay was performed in three independent biological experiments (*n* = 3). In each experiment, the full set of conditions was distributed across two assay plates because all conditions could not be accommodated on a single plate. Condition-matched tumor-cell survival controls and macrophage-only background controls were included on the corresponding assay plate in each experiment and were used for within-plate normalization of the matched co-culture conditions. Technical replicate wells were averaged within each experiment before calculation of tumor cell killing. For each condition, the condition-matched tumor-cell survival control was defined as B16-F10-luc2 cells treated with the same stimulus but cultured without macrophages (Supplementary Table S1). Thus, for VLP-treated conditions, the matched survival control consisted of B16-F10-luc2 cells treated with the corresponding VLP alone; for M1-associated conditions, B16-F10-luc2 cells treated with LPS/IFN-γ alone; and for M2-associated conditions, B16-F10-luc2 cells treated with IL-4 alone. For unstimulated control conditions, untreated B16-F10-luc2 tumor-cell-only wells were used as the survival control. Tumor cell killing was calculated as the percentage reduction in background-corrected luminescence relative to the matched survival control using the following formula:

$$\mathrm{Tumor}\;\mathrm{cell}\;\mathrm{killing}\;(\%)=\left[1-\frac{RLU_{co-culture,condition}-RLU_{macrophage-only,condition}}{RLU_{matched\;survival\;control,condition}-RLU_{macrophage-only,condition}}\right]\times 100$$

### 2.10. Luminex

Luminex assays were performed according to the manufacturer’s instructions using MILLIPLEX mouse cytokine/chemokine/growth factor panels (Millipore, USA; cat. no. MCYTOMAG-70K and MCYT1-190K). The MCYTOMAG-70K panel was used to analyze macrophage culture supernatants for IL-1β, IFN-γ, IL-6, IL-10, IL-12p40, and TNF-α, whereas the MCYT1-190K panel was used to analyze mouse sera for IL-1β, IFN-γ, IL-6, IL-10, IL-12p40, TNF-α, IL-17A, and IL-22.

For macrophage cytokine analysis, supernatants from two technical replicate culture wells were pooled to generate one sample per experimental condition. The 20 h collection time was selected as a standardized endpoint aligned with macrophage viability, morphology, and flow cytometric phenotyping; no cytokine time-course analysis was performed. The pooled samples were centrifuged at 500× *g* for 5 min, aliquoted, and stored at −70 °C until analysis. Each pooled sample was subsequently measured in duplicate in the Luminex assay. These duplicate measurements represent technical assay replicates rather than independent biological replicates. Accordingly, the macrophage cytokine data are presented descriptively as part of the comparative screening workflow.

Mouse sera from each VLP treatment group were pooled separately by sex and analyzed in three technical replicates. Macrophage supernatants and mouse serum samples were diluted 1:5 and 1:2, respectively, in assay buffer containing the appropriate matrix solution for serum samples. Samples were incubated overnight at 4 °C with agitation at 850 rpm in the dark in 96-well filter plates containing premixed magnetic beads, standards, and controls. The beads were washed twice and incubated with 25 µL of detection antibodies for 1 h at room temperature with agitation at 850 rpm in the dark. Subsequently, 25 µL of streptavidin–phycoerythrin was added, followed by incubation for 30 min under the same conditions. The plates were washed twice, the beads were resuspended in 150 µL of sheath fluid and shaken for 5 min, and fluorescence was measured using a Luminex 100/200^TM^ instrument with xPONENT software version 3.1.971.0 (Luminex Corporation, Austin, TX, USA).

### 2.11. Study Animals and In Vivo Experimental Design

The experimental procedures involving animals were approved by the National Animal Welfare and Ethics Committee (permit no. 158.2/2024) and were conducted in accordance with Directive 2010/63/EU as implemented in national legislation. In vivo procedures herein are reported in line with ARRIVE 2.0 guidelines to ensure a transparent and comprehensive description of animal use.

An individual animal served as an experimental unit in this study, and factorial randomized block design was applied (with 2 independent factors—the treatment (i.e., vaccine) and animal sex). Two animals were housed per single IVC to ensure randomization within 3 study blocks (procedures were blocked in time, with 2 days apart). Sample size was determined by power analysis using G*Power v3.1 software, assuming 80% power, α = 0.05, B16-F10 tumor growth dynamics variability (unpublished SD data from our previous study), the expected effect of a vaccine on tumor growth dynamics (changes in primary tumor size in time), and the planned primary outcome statistical tests, resulting in group sizes of eight animals for non-treated and mock (PBS) groups (4 males, 4 females) and twelve animals for each VLP treatment groups (6 males, 6 females; SrMV, PVM, CCMV-ss, RYMV, and CMVtt), yielding a total of 76 animals. Animals were assigned to study groups after simple randomization.

Naïve wild-type C57BL/6JOlaHsd male and female mice (10–11 weeks old; Envigo, Inotiv, Meterik, The Netherlands) with SPF status were used in all experiments and acclimatized for one week. Animals were assigned to cages after simple randomization, with two same-sex mice per cage. Two male animals had to be withdrawn from the study due to fighting injuries during the adaptation period. Mice were housed in IVCs (GM500, Tecniplast, Milan, Italy) connected to a SmartFlow air handling unit (Tecniplast) providing 75 air changes per hour in a positive pressure mode.

Animals had ad libitum access to autoclaved drinking water (acidified to pH 2.5–3.0 with HCl) and an autoclaved standard rodent maintenance diet (Mucedola, 4RF21 (A), Settimo Milanese, Italy). Autoclaved aspen bedding, nesting material (Tapvei, Paekna, Estonia), cardboard tunnels (Velaz, Prague, Czech Republic), and aspen gnawing blocks (Tapvei) were provided as environmental enrichment, and cages were changed every 10 days. Mice were maintained in an SPF facility at 24 ± 1 °C, 40–60% relative humidity, under a 12:12 h light–dark cycle (lights on 7:00–19:00), and health monitoring followed FELASA recommendations.

### 2.12. Tumor Experiments and Vaccination Regimen

During the acclimatization period prior to tumor implantation, two male mice (representing groups PVM and CCMV-ss) died from non-study-related causes and were excluded from the experiment, as stated in the methods section. All experimental animals were injected subcutaneously (s.c.) with 1 × 10^5^ B16-F10 cells (ATCC, Manassas, VA, USA) premixed 1:1 (*v*:*v*) with ice-cold Matrigel (Corning #356237, Corning, NY, USA) immediately prior to injection (total volume 50 µL) into the right flank of the animal under 2–3% isoflurane anesthesia.

Mice bearing palpable B16-F10 tumors of a palpable size ~150–200 mm^3^ were divided into seven groups (see Section 2.6 for graphic scheme): untreated (anesthesia only), mock (50 µL PBS), and 5 groups receiving different VLP vaccine treatment (25 µg VLPs in 50 µL PBS as prime dose and 50 µg VLPs in 50 µL PBS as booster dose). Treatments were administered intratumorally using 29 G needle under 2–3% isoflurane anesthesia on day 9 (for males) and day 11 (for females due to slower tumor growth) post-injection, with a second dose given 7 days later (i.e., day 16 for males, day 18 for females) using 50 µL PBS (mock), 50 µg VLPs in 50 µL PBS, or anesthesia only (untreated).

Mice were monitored daily for possible signs of suffering, and the primary tumor volumes were measured every 3 days using a digital calliper (Fine Science Tools, Foster City, CA, USA, cat. no. 30087-00) in a blinded fashion. The tumor volume was calculated using the formula *V*_T_ = (*L* × *W*^2^)/2, where *V* stands for tumor volume, *L*—length, and *W*—width. One female mouse from the PVM group died overnight prior to the scheduled endpoint procedure and was excluded from the final analysis. Animals were euthanized when tumors reached the ethically maximal size of 1.5 cm^3^ or experienced ulcerated lesions or showed signs of suffering defined by a suffering scoring sheet. Animals were humanely euthanized—under deep surgical isoflurane anaesthesia (5%)—and underwent cardiac puncture to collect terminal blood samples for serum isolation. Tumors were extirpated, weighed, and measured in three dimensions.

Tumors were then stored in DMEM containing 10% FBS on ice. Tumor-infiltrating lymphocytes (TILs) were isolated by first dissecting tumors into pieces, followed by enzymatic digestion with 2 mg/mL of collagenase D and 20 mkg/mL of DNAse in DMEM (37 °C, 30 min), and passage through a 70 µm cell strainer. Cells were washed with DMEM + 10% FBS in 50 mL Falcon tubes, resuspended in 15 mL tubes with 2 mL of 35% Percoll, and centrifuged at 800× *g* for 30 min at +10 °C to enrich TILs.

TILs were resuspended in 200 µL DMEM + 10% FBS, and 100 µL aliquots were transferred to duplicate U-bottom 96-well plates (one for extracellular, one for intracellular staining), then pelleted at 350× *g* for 5 min. Supernatants were discarded prior to antibody staining.

TILs were pelleted (350× *g*, 5 min, 10 °C), resuspended in 200 µL PBS, and blocked with TruStain FcX anti-mouse CD16/CD32 (BioLegend, cat. no. 101320) for 5 min in the dark. Cells were stained with Live/Dead Aqua fixable viability dye (Invitrogen, cat. no. L34957) for 30 min at 4 °C, washed, and split into two U-bottom 96-well plates.

Plate 1 (surface markers): Cells were stained for 30 min at 4 °C with PE/Cy7 CD45.2 (BioLegend, cat. no. 109830), eFluor660 CD3 (Invitrogen, cat. no. 50-164-26), FITC CD8α (BD Biosciences, San Jose, CA, USA, cat. no. 553031), V450 CD4 (BD Biosciences, cat. no. 560468), PE/Dazzle594 CD11b (BioLegend, cat. no. 101256), APC CD11c (AssayGenie, Dublin, Ireland, cat. no. AGEL0030), PerCP/Cy5.5 I-A/I-E (BioLegend, cat. no. 107626).

Plate 2 (intracellular markers): Cells were surface-stained for 30 min at 4 °C with FITC CD3 (Invitrogen, cat. no. 11-0032-82), PE/Cy7 CD8α (BioLegend, cat. no. 100722), and V450 CD4 (BD Biosciences, cat. no. 560468), fixed/permeabilized using BD Cytofix/Cytoperm Plus (cat. no. 554715), and stained for 30 min at 4 °C with PE/Dazzle594 Perforin (BioLegend, cat. no. 154316) and PerCP/Cy5.5 FoxP3 (Invitrogen, cat. no. 45-5773-82).

Samples were washed with FACS buffer (PBS + 2% FBS), acquired on a Cytek Aurora spectral flow cytometer (Cytek Biosciences, USA), and analyzed using FlowJo software suite (Tree Star, USA) and GraphPad Prism v8.4.2 software. Tumor-infiltrating lymphocyte (TIL) density was calculated as total TILs per tumor weight. To enable visualization of zero values, raw TIL density data were first transformed by addition of a constant (x + 0.1). The resulting values were then log_10_-transformed to improve data distribution and meet assumptions of normality. Statistical analyses were subsequently performed on the log_10_-transformed data.

### 2.13. Statistical Analysis

Quantitative data are presented as mean ± SEM where applicable. Group comparisons were analyzed using one-way ANOVA, whereas host sex × treatment interactions were analyzed using two-way ANOVA. Dunnett’s multiple-comparisons post hoc test was used for comparisons with the indicated control group. Pairwise differences were analyzed using unpaired Student’s *t*-test with Welch’s correction where appropriate. For post hoc comparisons of tumor endpoints, Dunnett’s test was performed against the non-treated anesthesia-only control to evaluate each intratumoral intervention relative to baseline tumor progression; the PBS group was included as a procedural/vehicle control for interpretation of injection-associated effects.

## 3. Results and Discussion

### 3.1. Endotoxin-Minimized Production and Physicochemical Characterization of Structurally Diverse Plant-Derived VLPs

To establish a standardized production platform with reduced endotoxin-related confounding, all eleven plant virus coat protein (CP) constructs were expressed in ClearColi BL21 (DE3), an engineered *Escherichia coli* strain with modified lipopolysaccharide (LPS) and markedly reduced endotoxic activity. This approach was selected to support downstream immunological comparisons while minimizing TLR4-mediated background effects. Previous work showed that Physalis mottle virus (PhMV) CP could be expressed in ClearColi without detectable alteration of VLP morphology or reduction in CP yield [24]. Bacteriophage P22 CP VLPs containing respiratory syncytial virus (RSV) matrix (M) proteins have likewise been successfully produced in ClearColi as well-defined particles of the expected size [25].

**Figure 1:**
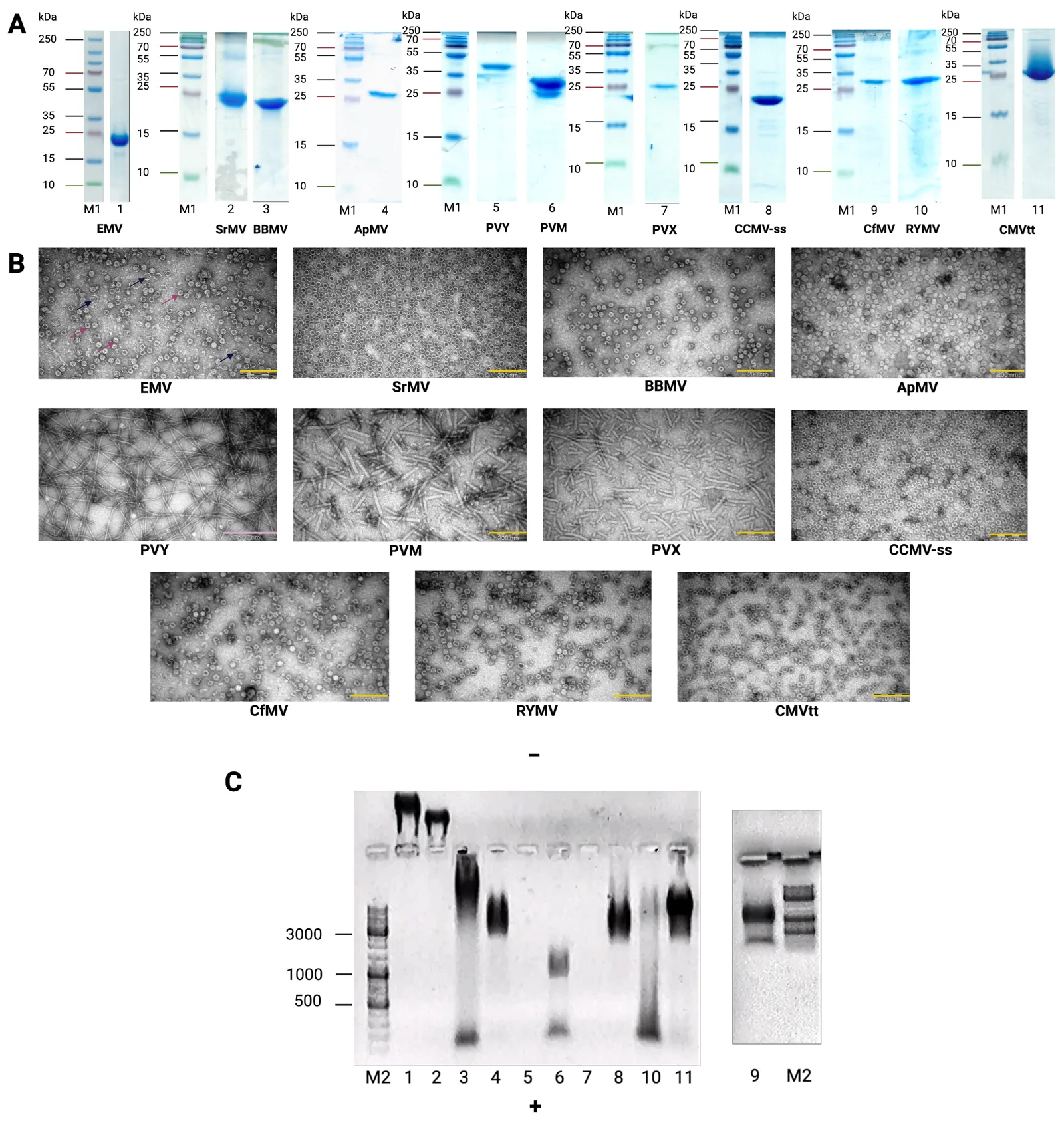
Analysis of ClearColi-expressed plant-derived coat protein (CP) virus-like particles (VLPs). (**A**) Coomassie—stained 12.5% SDS-PAGE of 1—EMV, 2—SrMV, 3—BBMV, 4—ApMV, 5—PVY, 6—PVM, 7—PVX, 8—CCMV-ss, 9—CfMV, 10—RYMV, 11—CMVtt at concentrations of 0.7–1 mg/mL. M1, PageRuler Plus Prestained Ladder, 10–250 kDa; the 70 kDa and 25 kDa reference bands are red, the 10 kDa band is green, the remaining bands are blue (ThermoFisherScientific, USA). (**B**) Electron micrographs of negatively stained VLPs. In the EMV micrograph, magenta arrows indicate representative intact VLPs, whereas blue arrows indicate open or incomplete structures of partially assembled particles. Scale bars—yellow (200 nm), pink (500 nm). (**C**) 0.8% native agarose gel analysis of ClearColi-expressed plant CP VLPs (1 mg/mL) stained with ethidium bromide. 1—EMV, 2—SrMV, 3—BBMV, 4—ApMV, 5—PVY, 6—PVM, 7—PVX, 8—CCMV-ss, 9—CfMV, 10—RYMV, 11—CMVtt. M2—GeneRuler DNA Ladder Mix (Thermo Fisher Scientific, USA, cat. no. SM0331). The symbols “+” and “−” denote the electrode polarity.

The panel included both filamentous and icosahedral plant VLPs. Seven constructs had been previously reported by our group—EMV [10], PVM [6], PVY [22], RYMV and CfMV [23], CCMV-ss [8], and CMVtt [7]—whereas the expression and VLP formation of SrMV, PVX, BBMV, and ApMV in *E. coli* are described here for the first time. CfMV and ApMV required co-transformation with pACYC-RIL to support efficient expression. All constructs yielded soluble CP and were characterized by SDS-PAGE, DLS, TEM, and 0.8% NAG electrophoresis (Figure 1). The principal production and physicochemical characterization outcomes are summarized in Table 1.

Both *Tymoviridae* family members, EMV and SrMV, yielded soluble CPs of approximately 20–21 kDa (Figure 1A; lanes 1 and 2), which assembled into T = 3 icosahedral particles of approximately 30 nm in diameter, consistent with previous descriptions [10]. Formation of mixed full and empty particles has previously been reported for *Tymoviridae* capsids and may be influenced by storage conditions and bacterial expression background [26]. In contrast, the newly expressed SrMV displayed a more homogeneous population of approximately 30 nm particles. NAG analysis indicated that both *Tymoviridae*-derived VLP preparations contained detectable nucleic acids. The observed signal may reflect incorporation of short ssRNA species that were not resolved as discrete bands under the native agarose gel conditions used here. Despite this nucleic acid signal, both VLP preparations migrated toward the negative electrode, indicating an overall net positive charge under the electrophoresis conditions used (Figure 1C, lanes 1 and 2). This electrophoretic behavior is likely explained by the relatively basic predicted isoelectric points of the EMV and SrMV CPs (pI = 8.52 and 8.91, respectively), together with retention of net positive charge by the assembled particles under the TBE electrophoresis conditions used. Although both VLP preparations contained detectable negatively charged nucleic acids, the amount of packaged nucleic acid was apparently insufficient to compensate for the positive charge of the CP assemblies.

The four *Bromoviridae*-derived constructs, BBMV, ApMV, CCMV-ss, and CMVtt, all yielded soluble CPs. BBMV and CCMV-ss showed apparent molecular weights of approximately 23 kDa (Figure 1A, lanes 3 and 8, respectively), whereas CMVtt migrated at approximately 27 kDa (Figure 1A, lane 11). ApMV has a theoretical molecular weight of approximately 24 kDa (Figure 1A, lane 4), but migrated at a slightly higher apparent molecular weight of approximately 27 kDa. This altered electrophoretic mobility is consistent with previous observations [6] and may be attributed to the high proline and glycine content of ApMV CP, as these residues can influence SDS-PAGE migration.

BBMV formed VLPs resembling those of CCMV-ss, consistent with their shared classification within the *Bromovirus* genus. In contrast, ApMV produced pleomorphic particles of heterogeneous sizes, including a mixture of T = 3 capsids and aggregated assemblies. These features likely reflect less optimal assembly conditions and altered electrostatic interactions during capsid formation, which can shift the equilibrium toward nonspecific aggregation [27].

The filamentous VLPs PVY and PVM also yielded soluble CP products, with apparent molecular weights of approximately 40 kDa and 35 kDa, respectively (Figure 1A, lanes 5 and 6). Both preparations showed additional bands in the approximately 25–35 kDa range, consistent with previously described proteolytic cleavage patterns for PVM and PVY CPs. These truncated CP variants are likely generated by trypsin-like enzymes in the *E. coli* system, such as oligopeptidase B, which recognize a homologous cleavage site, NKeKeK/DVN, within the CP sequence [6,9,22]. Although PVM has a theoretical molecular weight of approximately 35 kDa based on plasmid maps, its SDS-PAGE migration may be influenced by its high proline and glycine content, a feature known to affect electrophoretic mobility.

Expression of PVX CP in ClearColi yielded soluble protein with an apparent molecular weight of approximately 25 kDa (Figure 1A, lane 7), but the final yield was low, at approximately 0.3 mg/L. TEM revealed pronounced particle size heterogeneity, with some filaments measuring approximately 100–200 nm and others approximately 40–80 nm in length (Figure 1B). This heterogeneous assembly is consistent with the known RNA-dependent self-assembly of PVX CP, which typically requires a specific nucleation site, stem-loop 1, to form extended filaments; in the absence of this scaffold, the protein may preferentially form shorter assemblies or aggregates [28].

Among the filamentous constructs, only PVM showed detectable nucleic acid incorporation by NAG (Figure 1C, lane 6), whereas PVY and PVX showed no detectable signal (Figure 1C, lanes 5 and 7). This result is consistent with previous findings that filamentous VLPs typically encapsulate a very low percentage of nucleic acid (~3%) within their VLPs [6], often falling below the detection threshold of standard staining methods.

The expected molecular weights of the *Solemoviridae* family members CfMV and RYMV were approximately 27 kDa and 30 kDa, respectively (Figure 1A, lanes 9 and 10). Compared with previous expression in the BL21 (DE3) system, where successful T = 3 VLP formation was reported [23], expression in ClearColi was associated with reduced assembly quality for both constructs. RYMV formed several pleomorphic particles (Figure 1B), suggesting that the ClearColi expression background may not fully support the specific structural requirements of these sobemovirus capsids, which rely on calcium ions and pH-dependent interactions to maintain icosahedral symmetry [29]. RYMV predominantly packaged short RNA fragments together with a minor fraction of larger nucleic acids, whereas CfMV encapsulated larger RNA species, appearing as a distinct band between approximately 1000 and 3000 bp (Figure 1C, lane 9).

Collectively, these data show that ClearColi BL21 (DE3) supported soluble expression of all eleven plant-derived CP constructs, although yield, assembly fidelity, morphology, and nucleic acid incorporation varied substantially across VLPs. This standardized endotoxin-minimized production and characterization workflow enabled direct comparison of structurally diverse plant-derived VLP biomaterials and provided the basis for subsequent immune-functional screening and in vivo antitumor evaluation.

### 3.2. Dose-Dependent Cytocompatibility of Plant-Derived VLPs in RAW264.7 Macrophages

**Figure 2:**
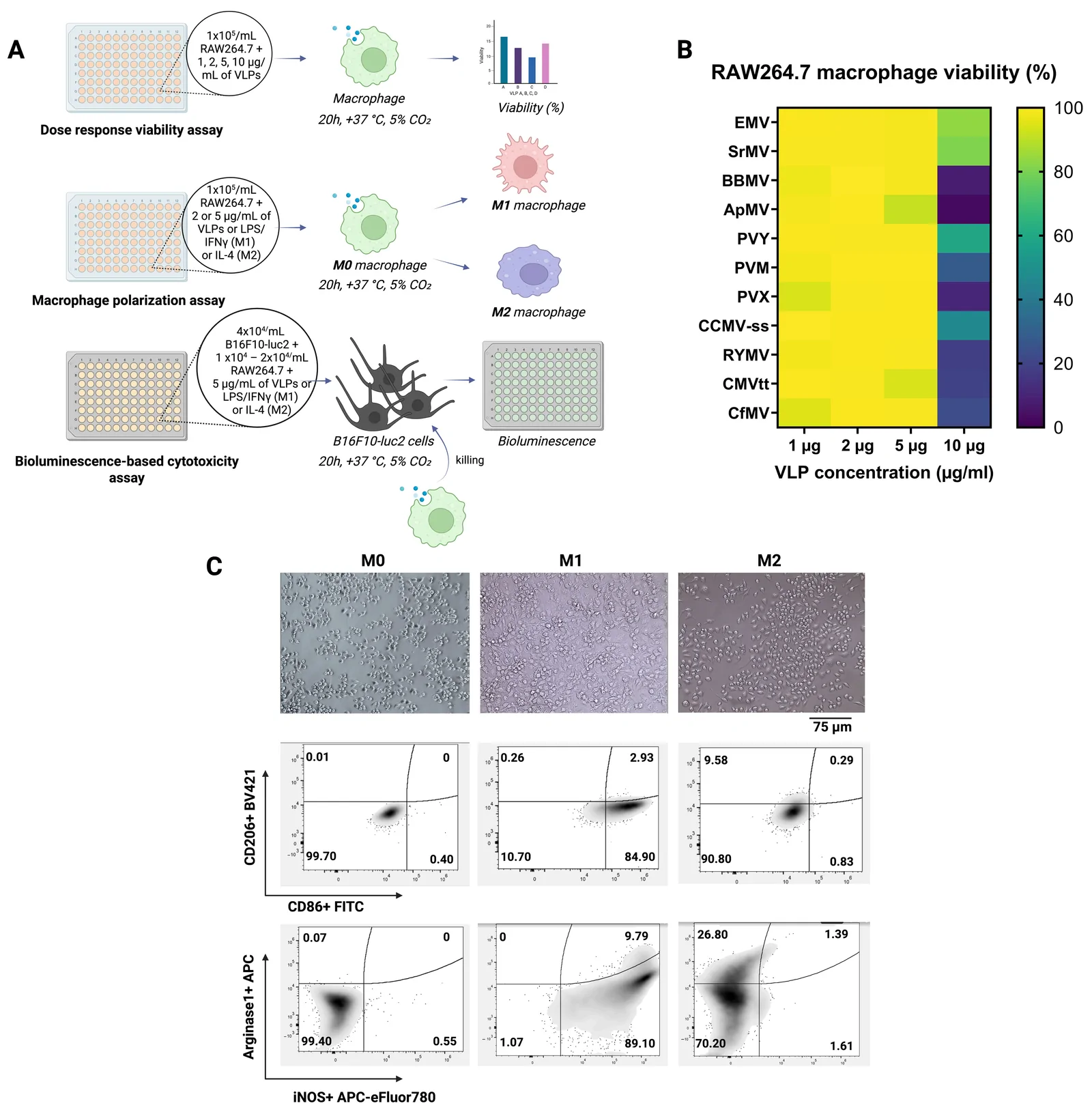
Experimental in vitro workflow, plant VLP cytotoxicity assessment, and macrophage activation controls. (**A**) Schematic overview of the experimental in vitro workflow. RAW264.7 macrophages were seeded at 1 × 10^5^ cells/mL and stimulated with increasing concentrations of plant virus-like particles (VLPs) for dose–response viability assessment. For macrophage polarization assays, cells were treated with VLPs or classical stimuli (LPS/IFN-γ for M1 and IL-4 for M2) and incubated for 20 h at 37 °C and 5% CO_2_. For functional assessment, polarized macrophages were co-cultured with B16-F10-luc2 melanoma cells, and tumor cell killing was quantified using a bioluminescence-based cytotoxicity assay. (**B**) Heatmap summarizing RAW264.7 macrophage viability following 20 h stimulation with increasing concentrations (1–10 µg/mL) of the indicated plant-derived VLPs, expressed as the percentage of live cells as determined by flow cytometric viability staining. Data represent pooled flow cytometry samples generated from two technical replicate wells per condition. The heatmap is presented descriptively to illustrate relative viability trends across the VLP panel, establishing the dose-dependent profile for each VLP candidate. (**C**) Representative phase-contrast microscopy images (**top**; scale bar, 75 µm) and flow cytometric analysis (**bottom**) of macrophages under M0 (unstimulated), M1 (LPS/IFN-γ), and M2 (IL-4) control conditions. Flow cytometry plots show surface expression of CD206 and CD86 (**top row**) and intracellular expression of arginase-1 and iNOS (**bottom row**), with quadrant values indicating the percentage of cells in each population.

To support side-by-side biomaterial screening and reduce unnecessary reliance on animal tumor models during early candidate selection, we established a cell culture-based comparison workflow to evaluate plant-derived VLPs as candidate in situ cancer immunotherapy platforms (Figure 2A). As an initial step, we assessed whether the VLP preparations affected the viability of RAW264.7 murine macrophages, which were selected as a macrophage model for subsequent immune-functional assays. Two technical replicate wells were prepared per condition and pooled before flow cytometric acquisition. The data are presented descriptively to illustrate relative viability trends across the VLP panel for candidate-selection purposes.

To minimize nonspecific stimulation, all VLPs used in these assays were produced in the ClearColi^TM^ system. Residual endotoxin levels were maintained below 100 EU/mg protein, ensuring that the final endotoxin concentration in the culture medium did not exceed 1 EU/mL at the highest VLP dose tested. This enabled cytocompatibility and immune-functional screening under endotoxin-minimized conditions, although a contribution from trace endotoxin cannot be fully excluded.

Across all eleven VLPs tested, macrophage viability remained largely unaffected at concentrations of 1, 2, and 5 μg/mL, with live cells exceeding 90% for all samples (Figure 2B). In contrast, a marked reduction in viability was observed at 10 μg/mL, particularly for BBMV and ApMV VLPs. These findings indicate that most VLP preparations were cytocompatible with RAW264.7 macrophages within the lower concentration range, whereas higher concentrations induced construct-dependent reductions in macrophage viability.

The reduced viability observed at 10 μg/mL may reflect increased immune activation rather than direct VLP-associated cytotoxicity. Previous studies have shown that the immunostimulatory activity of VLPs can be influenced by encapsidated prokaryotic or viral ssRNA, which may engage endosomal receptors such as TLR7 following cellular uptake [30,31]. However, the relationship between detectable nucleic acid content and macrophage viability was not uniform across the panel. For example, EMV and SrMV showed detectable nucleic acid signal by NAG, yet maintained comparatively higher viability at the highest VLP dose. This may reflect differences in RNA amount, length, accessibility, sequence composition, particle uptake, or intracellular processing, all of which could influence the magnitude of innate immune activation.

These observations are consistent with previous studies suggesting that RNA-associated properties can influence the immunostimulatory activity of VLPs [30,32]. However, the present viability screen does not establish a direct correlation between the nucleic acid signal observed in Figure 1C and macrophage viability responses across the VLP panel. Instead, detectable nucleic acid content was considered as one particle-associated feature among others, including morphology, assembly quality, surface organization, uptake, and intracellular processing, that may contribute to candidate-specific immune-functional profiles. Nevertheless, the specific contribution of TLR7 signaling was not directly assessed in the present study. Based on the viability profile across the panel, 2 and 5 μg/mL were selected as cytocompatible working concentrations for subsequent in vitro immune-functional assays.

### 3.3. Comparative Evaluation of Plant-Derived VLPs Reveals Divergent Macrophage Activation Profiles

Classically activated macrophages can contribute to antitumor immunity through phagocytosis, antigen presentation, and production of pro-inflammatory mediators [33,34]. Therefore, after establishing cytocompatible working concentrations, we next evaluated whether the plant-derived VLP panel could modulate macrophage activation in RAW264.7 cells (Figure 2A). Rather than defining fixed macrophage polarization states, we assessed whether VLP stimulation induced M1- or M2-associated marker patterns using LPS/IFN-γ as an M1-like activation reference and IL-4 as an M2-associated activation reference (Figure 2C). Activation status was evaluated by light microscopy and by measuring expression of M1-associated markers, CD86 and iNOS, and M2-associated markers, CD206 and Arginase-1. The M2-associated marker arm was included not because alternative macrophage activation was expected to mediate antitumor activity, but to determine whether any VLP candidate promoted an alternative, potentially tumor-supportive macrophage phenotype. This was relevant for candidate evaluation, as intratumoral immunotherapy candidates should ideally promote inflammatory immune activation without inducing a dominant M2-associated marker profile.

Because the RAW264.7 phenotyping and macrophage cytokine experiments were designed as comparative screening assays, the corresponding data should be interpreted as descriptive trends supporting candidate selection rather than as independently validated mechanistic findings.

As expected, unstimulated M0 macrophages displayed a low-activation phenotype characterized by rounded morphology (Figure 2C). Flow cytometry analysis showed minimal expression of activation markers in this group, with most cells remaining double-negative for both surface and intracellular marker combinations, CD206^−^CD86^−^ and Arginase-1^−^iNOS^−^ (Figure 2C). In contrast, stimulation with LPS/IFN-γ induced pronounced morphological changes after 20 h, resulting in enlarged, irregular, amoeboid cells with visible vacuole-like structures and pseudopodia [35,36]. Consistent with these observations, flow cytometry showed increased expression of CD86, approximately 85%, and a distinct iNOS-positive population, approximately 89%, indicative of an M1-associated activation profile.

M2-associated activation was less pronounced under these conditions. Following IL-4 stimulation, RAW264.7 macrophages exhibited heterogeneous morphology, with a mixture of rounded cells and a smaller fraction of elongated, spindle-shaped cells characteristic of M2-associated activation (Figure 2C). Flow cytometric analysis supported this limited response, with only approximately 10% of cells displaying a CD206^+^CD86^−^ phenotype, while the majority remained negative for both surface markers. In contrast, intracellular staining showed that approximately 27% of cells were Arginase-1^+^iNOS^−^, suggesting partial induction of M2-associated metabolic features. Thus, the IL-4 condition did not induce a robust or uniform M2-associated phenotype in RAW264.7 cells under the present experimental conditions. For this reason, the M1/M2-associated marker panel was used as a reference framework for comparing relative activation trends rather than as definitive evidence of complete macrophage polarization.

**Figure 3:**
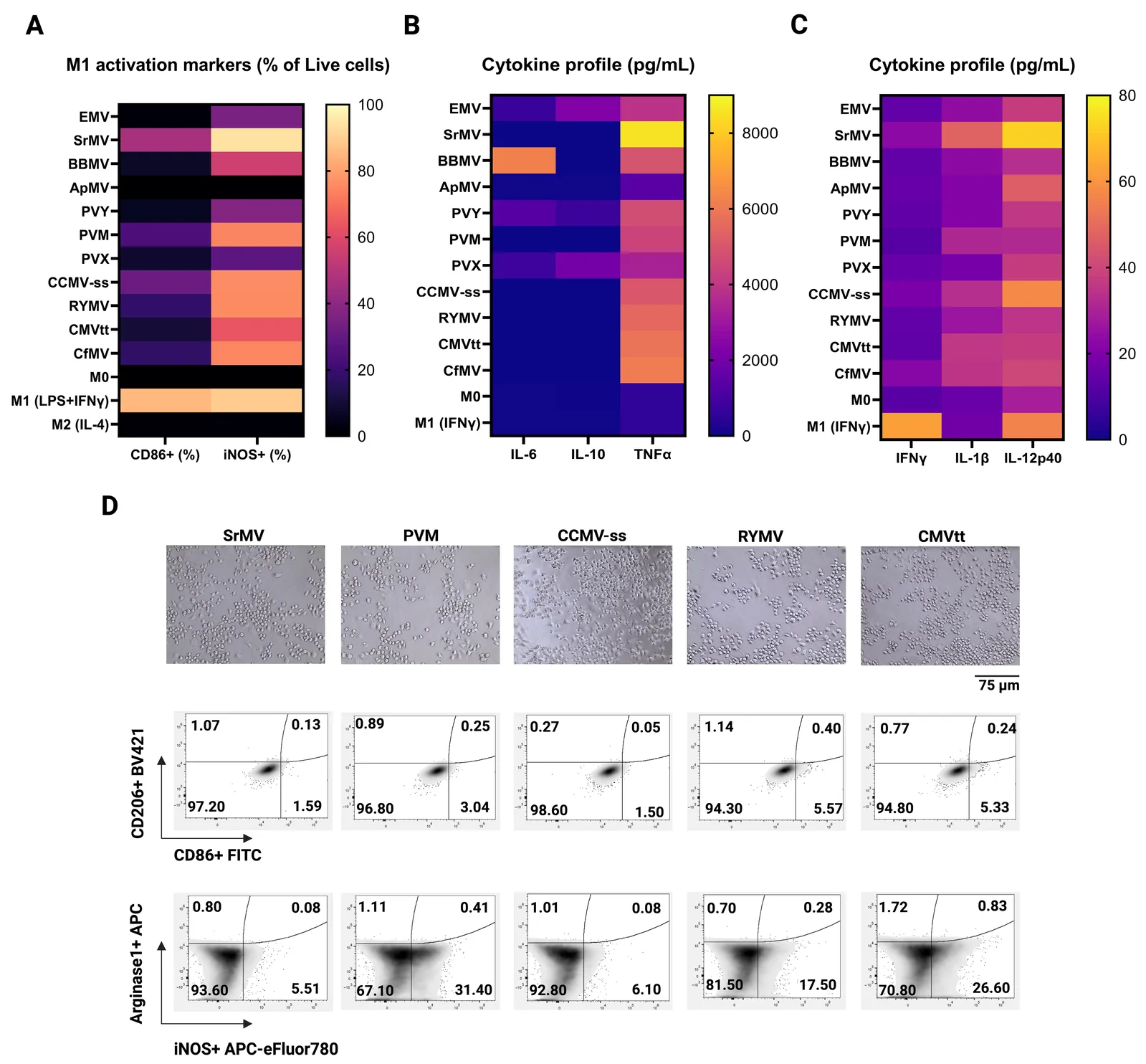
Comparative screening of macrophage responses to plant virus-like particles (VLPs). (**A**) Heatmap summarizing M1-associated surface and metabolic marker expression, shown as the percentage of live cells positive for CD86 and iNOS following 20 h stimulation of macrophages with 11 plant-derived VLPs in 96-well plates. M0 macrophages and M1-associated (LPS + IFN-γ) and M2-associated (IL-4) reference controls are included for comparison. (**B**) Cytokine secretion profile of macrophages stimulated with the indicated VLPs, measured in culture supernatants and displayed as concentrations (pg/mL) of IL-6, IL-10, and TNF-α. (**C**) Additional cytokine profiling showing IFN-γ, IL-1β, and IL-12p40 levels (pg/mL) under the same screening conditions. (**D**) Representative phase-contrast microscopy images (**top**; scale bar, 75 µm) and flow cytometric analysis (**bottom**) of macrophages stimulated with five selected VLP candidates (SrMV, PVM, CCMV-ss, RYMV, and CMVtt). Flow cytometry plots show the surface expression of CD86 and CD206 (**top row**) and the intracellular expression of iNOS and Arginase-1 (**bottom row**), with quadrant values indicating the percentage of cells within the respective populations. Flow cytometry and cytokine data were generated from technical duplicate wells pooled before analysis and are presented descriptively as part of the comparative screening workflow.

We next assessed the ability of all eleven plant-derived VLPs, at 2 μg/mL, to induce inflammatory macrophage activation under these screening conditions. Overall, most VLPs induced low to moderate surface expression of CD86 while promoting higher intracellular iNOS expression (Figure 3A). Among the tested candidates, SrMV elicited the strongest response, with approximately 46% CD86^+^ cells and 94% iNOS^+^ cells. PVM and CCMV-ss also induced notable responses, with moderate CD86 expression, approximately 24% and 32%, respectively, and robust iNOS induction, approximately 75% and 76%, respectively. In contrast, RYMV, CMVtt, and CfMV showed more modest surface marker expression while still inducing measurable iNOS levels. ApMV did not induce appreciable expression of M1-associated markers under these conditions. These data suggest that several VLP biomaterials can promote inflammatory activation of macrophages, although the magnitude of surface and intracellular marker responses varied substantially across candidates. Importantly, none of the tested VLPs induced a clear M2-associated marker pattern, indicating that the selected VLPs did not promote detectable alternative macrophage skewing under these screening conditions.

To further assess functional activation, cytokine production was analyzed using a Luminex multiplex assay (Figure 3B,C). Supernatants from VLP-treated samples, as well as M0 controls and IFN-γ-primed controls, were evaluated for six cytokines associated with macrophage activation: IL-6, IL-10, TNF-α, IFN-γ, IL-1β, and IL-12p40. IFN-γ priming alone resulted in limited cytokine induction, with elevated IL-12p40 but minimal TNF-α and IL-1β production. In contrast, several VLPs were associated with broader cytokine accumulation at the 20 h endpoint, consistent with macrophage activation under these screening conditions. Among the tested candidates, SrMV induced the most pronounced cytokine profile, characterized by elevated TNF-α, IL-12p40, IL-6, and IL-1β levels. CCMV-ss and PVM also elicited pro-inflammatory cytokine patterns, whereas ApMV, EMV, and PVY showed minimal cytokine induction. These findings are consistent with the flow cytometry data and indicate that selected VLPs can promote inflammatory macrophage activation within this experimental system.

Importantly, these assays do not distinguish between direct VLP-induced effects and downstream macrophage-mediated responses. Therefore, the data should be interpreted as reflecting combined system-level activity under the tested in vitro conditions. Based on the overall trends observed across marker expression and cytokine production, SrMV, PVM, and CCMV-ss were prioritized for further analysis, together with RYMV and CMVtt, which showed moderate responses and represented structurally distinct VLP candidates.

To further characterize these five candidates, macrophages were stimulated under modified culture conditions in 24-well plates at 5 μg/mL. Under these conditions, reduced surface and intracellular marker expression was observed compared with the 96-well format. This likely reflects differences in culture format, including cell density, medium volume, surface area, and local particle-to-cell exposure, all of which can influence macrophage activation [37]. Morphological analysis revealed predominantly M0-like cells with only occasional M1-associated features (Figure 3D). Flow cytometry confirmed that most cells remained CD206^−^CD86^−^, with only modest CD86 induction in a small fraction of cells. Intracellular analysis showed moderate iNOS induction by PVM, RYMV, and CMVtt. These results suggest that, under less stimulatory culture conditions, selected VLPs promote partial inflammatory priming rather than full macrophage polarization.

Macrophage activation within the TME has traditionally been stratified into two opposing states: classically activated M1 macrophages and alternatively activated M2 macrophages [38]. However, this binary framework largely represents an in vitro simplification [39]. In vivo, tumor-associated macrophages (TAMs) are highly heterogeneous and exhibit substantial phenotypic and functional diversity, rarely conforming to rigid M1 or M2 identities [39,40]. Consequently, reliance on canonical M1/M2 markers alone may not fully predict functional antitumor activity or therapeutic outcomes in vivo. Overall, these findings support the use of the marker panel as a comparative screening tool, while also demonstrating that classical M1/M2-associated marker expression alone was insufficient to define functionally relevant macrophage states. Therefore, we selected five structurally and functionally distinct plant-derived VLP candidates, SrMV, PVM, CCMV-ss, RYMV, and CMVtt, for further assessment of their immunomodulatory and antitumor potential.

### 3.4. Plant-Derived VLPs Enhance Macrophage-Associated Tumor Cell Killing in a Ratio-Dependent Manner

Macrophage-associated tumor control, including cytotoxic activity, phagocytosis, and contact-dependent killing, can occur independently of classical M1-associated marker expression and may be shaped by the activation state and local microenvironment [41]. Therefore, functional assays are necessary to evaluate antitumor activity beyond marker-based macrophage classification.

**Figure 4:**
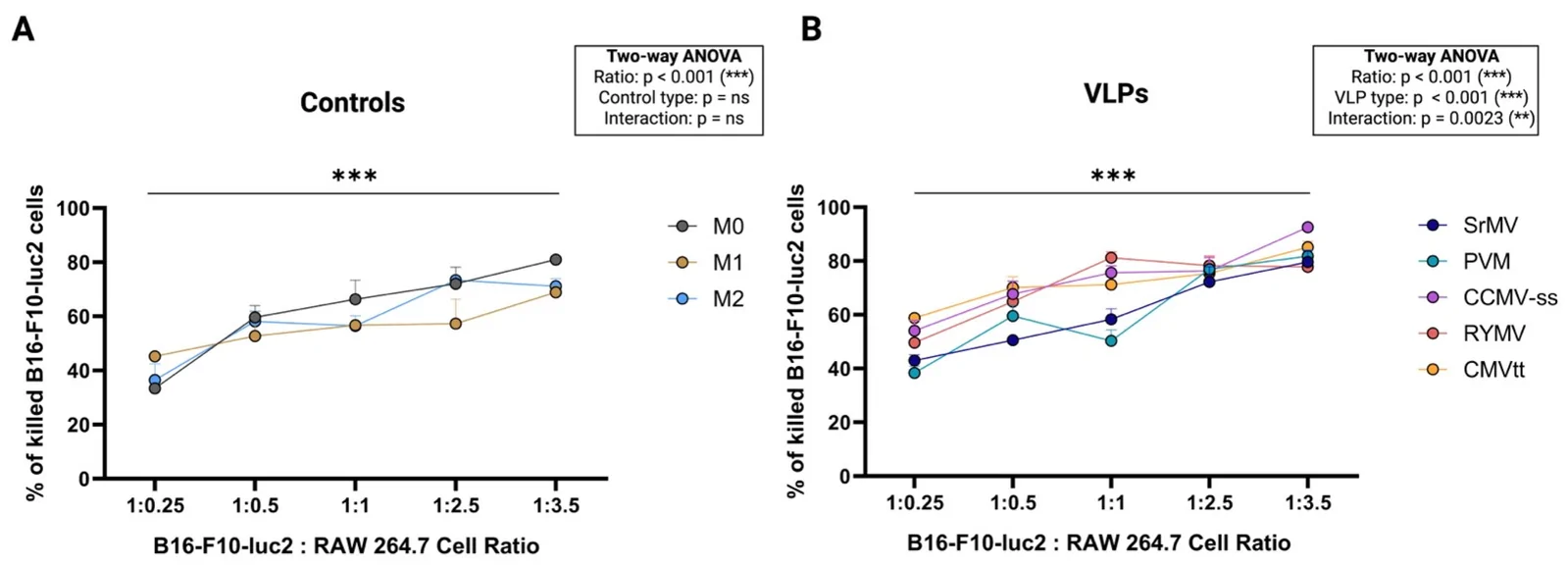
Macrophage-associated tumor cell killing in co-culture assays. (**A**) Percentage of B16-F10-luc2 melanoma cells (4 × 10^4^ cells/mL) killed after 20 h co-culture with RAW264.7 macrophages under M0, unstimulated; M1-associated, IFN-γ/LPS; or M2-associated, IL-4; conditions at the indicated macrophage-to-tumor cell ratios. (**B**) Percentage of B16-F10-luc2 melanoma cells killed following co-culture with RAW264.7 macrophages treated with plant-derived VLPs (5 µg/mL). Data are shown as mean ± SEM from three biological replicates; statistical significance was assessed by two-way ANOVA and is indicated in the figure; ns, not significant; ** *p* < 0.01; *** *p* < 0.001.

To assess macrophage-associated antitumor activity, we performed a co-culture assay using B16-F10-luc2 murine melanoma cells and RAW264.7 macrophages under resting, M0; pro-inflammatory, M1-associated; or alternative, M2-associated; conditions, as well as in the presence of five selected plant-derived VLPs: SrMV, PVM, CCMV-ss, RYMV, and CMVtt. A two-way ANOVA revealed that the macrophage-to-tumor cell ratio was the primary driver of cytotoxicity in both control and VLP-treated groups (*p* < 0.001). In control conditions (Figure 4A), the macrophage activation state (M0, M1-associated, or M2-associated) did not exert a statistically significant influence on killing efficiency (*p* = ns). At the lowest macrophage-to-tumor cell ratio, 1:0.25, M1-associated macrophages showed the highest cytotoxic activity among the control conditions, achieving more than 40% tumor cell killing. However, at higher macrophage ratios, 1:0.5–1:3.5, unstimulated M0 macrophages showed greater tumor cell killing, reaching approximately 60–80% cytotoxicity. In contrast, M1-associated macrophages showed only limited increases in cytotoxicity with increasing macrophage ratios, suggesting that pro-inflammatory activation, as defined by canonical markers, did not directly translate into enhanced tumor cell killing under these assay conditions.

This response pattern is consistent with previous reports showing that classically activated M1-associated macrophages can rely on soluble cytotoxic mediators, such as nitric oxide (NO) and reactive oxygen species (ROS), which may be associated with reduced phagocytic capacity under certain conditions [42]. In contrast, resting M0 and alternative M2-associated macrophages may retain greater phagocytic or contact-dependent killing capacity in selected experimental contexts [43]. Thus, marker-defined macrophage activation states may not directly predict functional tumor cell killing.

In the presence of selected plant-derived VLPs (Figure 4B), a two-way ANOVA indicated a statistically significant interaction between VLP type and cell ratio (*p* = 0.0023). While high macrophage ratios (1:3.5) consistently achieved high cytotoxicity (70–90%) across all groups, VLP-specific differences were most apparent at lower macrophage-to-tumor cell ratios. Among the tested VLPs, CMVtt showed the highest tumor cell killing at the lowest macrophage-to-tumor cell ratios, 1:0.25 and 1:0.5, reaching approximately 60–70% cytotoxicity. This may indicate enhanced per-cell cytotoxic activity under these conditions; however, the underlying mechanisms were not directly assessed. Potential contributors could include soluble mediators, such as mentioned NO, ROS [42], or cytokine-dependent effects, although these remain speculative in the context of the present study.

All VLP-treated macrophage conditions exhibited ratio-dependent tumor cell killing, with the largest increases generally observed between low and intermediate macrophage-to-tumor cell ratios, 1:0.25 to 1:1 (Figure 4B). Across several VLPs, including CCMV-ss, RYMV, and CMVtt, cytotoxicity appeared to plateau around a 1:1 ratio, suggesting possible saturation of killing capacity under these assay conditions. Although filamentous PVM VLPs showed increased variability at intermediate ratios, 1:0.5 and 1:1, the overall trend of increasing tumor cell killing with higher macrophage ratios was maintained.

In immunocompetent tumor models, macrophage activation and function are influenced by interactions with other immune cells, particularly lymphocytes, through cytokine-mediated signaling [44,45]. These interactions can substantially alter macrophage behavior and therapeutic outcomes, highlighting that macrophage activity in tumors cannot be fully recapitulated in isolated in vitro co-culture systems [46]. Nevertheless, these results demonstrate that selected plant-derived VLP biomaterials can enhance macrophage-associated antitumor activity in vitro and support their further evaluation in immunocompetent melanoma models.

### 3.5. Comparative TLR3 Reporter Activation by Selected Plant-Derived VLPs

Activation of TLR signaling contributes to early innate immune responses and downstream inflammatory activity [47,48]. TLR3 was originally characterized as a sensor of double-stranded RNA, as demonstrated using the synthetic analog polyinosinic–polycytidylic acid, poly(I:C), which mimics viral nucleic acids and induces antiviral innate immune responses through production of type I interferons and inflammatory cytokines [14]. Although VLP-associated RNA may also engage other endosomal RNA sensors, including TLR7 [49], only TLR3-associated activity was directly assessed in the present study.

To complement the macrophage-based assays, five selected VLPs—CMVtt, CCMV-ss, SrMV, RYMV, and PVM—were evaluated using HEK-Blue^TM^ hTLR3 reporter cells [50]. The candidates were selected based on their structural diversity and immune-functional profiles. Because the preparations contained different amounts of associated RNA, intact VLP inputs were normalized to RNA content to permit comparison at RNA-equivalent doses.

**Figure 5:**
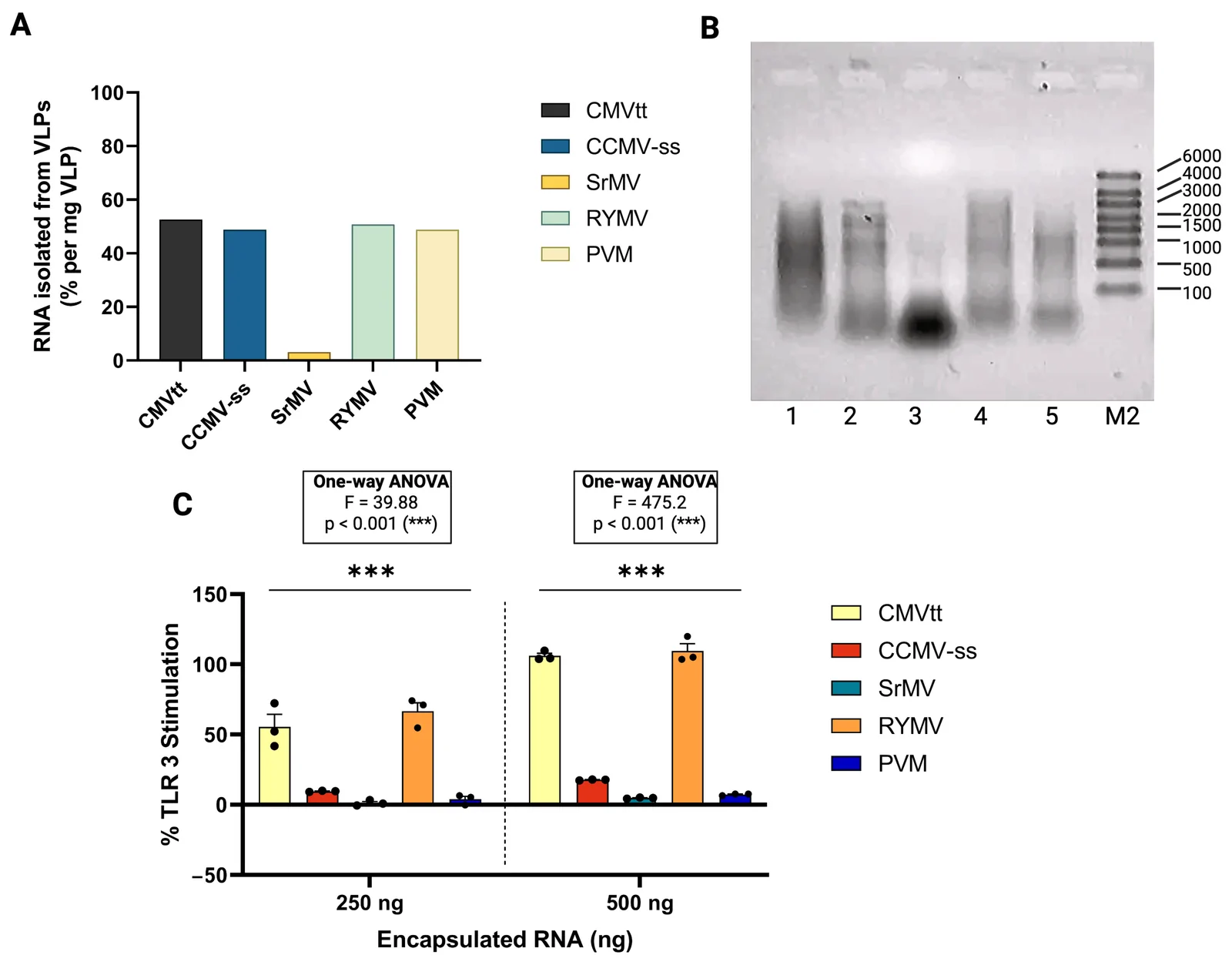
RNA content of VLP preparations and TLR3 activation by intact particles. (**A**) Total RNA isolated from five different plant virus-like particles (VLPs), expressed as a percentage relative to 1 mg of VLP protein. VLPs analyzed were CMVtt, CCMV-ss, SrMV, RYMV, and PVM. (**B**) Encapsidated RNA isolated from five different VLPs was analyzed by 1% agarose gel electrophoresis. Lanes correspond to: (1) CMVtt, (2) CCMV-ss, (3) SrMV, (4) RYMV, and (5) PVM. The RNA size marker (M2) is shown on the right, with fragment sizes indicated in base pairs (bp). A total of 400 ng of RNA was loaded per lane. (**C**) Activation of TLR3 signaling in HEK-Blue^TM^ hTLR3 reporter cells following stimulation with intact VLPs containing 250 ng or 500 ng of encapsidated RNA. TLR3 stimulation is presented as a percentage relative to the control stimulated with poly(I:C). Data are shown as mean ± SEM with individual data points indicated. Statistical significance was determined by one-way ANOVA (F = 39.88 for 250 ng; F = 475.2 for 500 ng; *** *p* < 0.001).

RNA isolation revealed differences in both RNA content and apparent size distribution among the selected VLP preparations (Figure 5A,B). CMVtt, CCMV-ss, RYMV, and PVM contained broadly comparable RNA levels, approximately 45–55% *w*/*w* relative to VLP protein, whereas SrMV contained substantially less RNA. CMVtt showed a prominent population of larger RNA species of approximately 1–2 kb, SrMV predominantly contained shorter fragments below 100 bp, and CCMV-ss, RYMV, and PVM displayed more heterogeneous RNA profiles.

Despite RNA-normalized stimulation, the intact VLP preparations induced markedly different TLR3 reporter responses (Figure 5C). CMVtt induced 55.42% activation at 250 ng RNA and 106.03% at 500 ng RNA, relative to the poly(I:C) control defined as 100%. RYMV similarly induced strong activation, reaching 66.61% and 109.51% at 250 and 500 ng RNA, respectively. In contrast, CCMV-ss induced only 9.49% and 17.69% activation, while SrMV showed no detectable activation at 250 ng RNA and only 4.70% activation at 500 ng RNA. PVM also showed minimal activation under the same conditions. Notably, CCMV-ss and RYMV produced markedly different reporter responses despite broadly overlapping apparent RNA size profiles. Thus, TLR3 reporter activation was not predicted solely by total RNA content or apparent RNA size distribution.

Overall, the HEK-Blue^TM^ hTLR3 reporter assay provided an exploratory comparative readout of RNA-associated immunostimulatory activity among the selected intact VLP preparations. Although CMVtt and RYMV induced the strongest responses, whereas SrMV, CCMV-ss, and PVM showed limited activation under RNA-normalized conditions, the present data do not identify the molecular features responsible for these differences. Because intact VLP preparations were tested, potential contributions from RNA sequence or secondary structure, RNA accessibility, particle uptake and endosomal processing, or other preparation-associated components cannot be distinguished by the current experimental design. Future studies using nuclease-sensitivity assays, RNA-depleted or disrupted particles, targeted RNA sequence and structural analyses, and complementary reporter systems, including TLR7 assays, will be required to define the relevant immunostimulatory features and sensing pathways.

### 3.6. Tumor–Host Sex Context Is Associated with Tumor Progression and Response to VLP-Based Intratumoral Treatment in the B16-F10 Melanoma Model

Appropriate experimental models are essential for cancer research and therapeutic development, as they should recapitulate key features of the TME. While two-dimensional cell culture systems are widely used for early screening, they do not capture tumor architecture, cellular heterogeneity, stromal interactions, or microenvironmental gradients [51]. In contrast, syngeneic mouse models, such as B16-F10 melanoma in C57BL/6 mice, allow tumor growth in immunocompetent hosts. However, B16-F10 is a male-derived melanoma cell line; therefore, implantation into female mice represents a sex-mismatched tumor–host setting [52]. This is an important consideration when interpreting host sex-associated outcomes in this model.

Because B16-F10 is male-derived, differences in tumor progression observed in female recipients cannot be attributed to host sex alone. Implantation of male-derived tumor cells into female mice introduces a tumor–host sex mismatch and may enhance immune recognition through male-specific minor histocompatibility antigens, including H-Y antigens. The present study was not designed to quantify the relative contributions of H-Y antigen recognition and other host sex-associated biological factors. Therefore, comparisons between male and female recipients should be interpreted as reflecting tumor–host sex context rather than isolated host sex effects.

Melanoma is a highly aggressive skin cancer with broadly comparable incidence between sexes at younger ages but higher mortality in men later in life [53,54]. Despite melanoma being one of the most extensively studied cancers in immunotherapy research, many preclinical studies remain biased toward the use of female animals. Numerous immunotherapy studies employing melanoma models have been conducted exclusively in female mice [4,55,56,57], even though males represent the population with the highest disease burden. In addition, sex-associated differences in melanoma progression have been reported previously. For example, Dakup and colleagues [58] showed that the B16-F10/C57BL/6 model can reflect aspects of melanoma progression observed in humans and reported stronger antitumor immune responses in females than in males.

**Figure 6:**
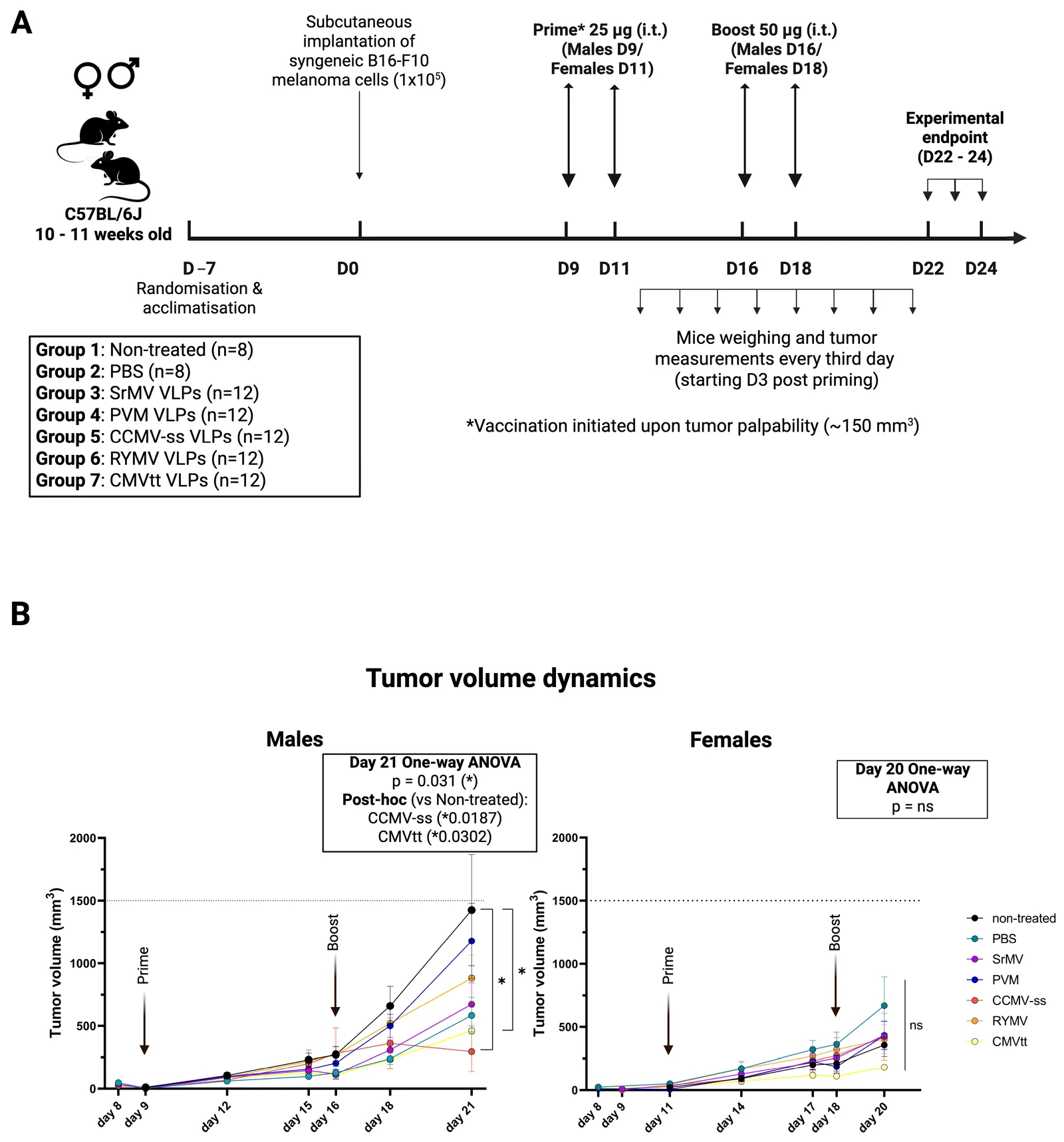
Experimental design and tumor growth following intratumoral administration of plant-derived VLPs in the B16-F10 melanoma model. (**A**) Experimental design and treatment schedule. Male and female C57BL/6J mice were subcutaneously injected with B16-F10 melanoma cells and vaccinated intratumorally with plant VLPs following tumor palpability. Tumor growth and body weight were monitored throughout the study. (**B**) Tumor-volume dynamics in male and female mice. Data are presented as mean ± SEM. Treatment-group comparisons were performed separately for each sex using one-way ANOVA with post hoc comparisons against the non-treated group, as indicated. * *p* < 0.05; ns, not significant.

In the present study, we included both male and female C57BL/6J mice by first assessing B16-F10 tumor progression following subcutaneous injection and then evaluating host sex-associated responses to plant VLP-based intratumoral treatment administered as two local in situ vaccinations. Experiments were conducted according to the timeline shown in Figure 6A and included seven groups: a non-treated control, a PBS injection control, and five VLP treatment groups, SrMV, PVM, CCMV-ss, RYMV, and CMVtt. Treatment was initiated when tumors became palpable, on day 9 in males and day 11 in females, and this timing difference should be considered when comparing tumor progression between sexes (Figure 6A).

Following the first intratumoral treatment, tumor growth was broadly comparable between sexes, with mean tumor volumes in all groups remaining below 250 mm^3^ until days 14–15 (Figure 6B). After the booster treatment, more pronounced treatment-associated differences emerged in male mice. By day 18, tumors in the non-treated, PVM, and RYMV groups had reached approximately 500 mm^3^, whereas the CCMV-ss-treated group showed a modest delay in tumor growth (Figure 6B). This effect became more pronounced by day 21, when the CCMV-ss group displayed the smallest mean tumor volume among the male treatment groups.

At endpoint, four of seven male groups reached mean tumor volumes of approximately 500 mm^3^ or higher, with the largest tumors observed in the non-treated control group, approaching 1500 mm^3^. After two intratumoral treatments, a significant reduction in tumor volume was observed on day 21 in male mice treated with CCMV-ss and CMVtt VLPs compared with non-treated controls (Figure 6B; *p* = 0.0187 and *p* = 0.0302, respectively), consistent with treatment-associated delay of tumor progression in males.

In female mice, no statistically significant differences between treatment groups were observed despite continued tumor progression (Figure 6B). By day 20, only the PBS-treated group reached a mean tumor volume of approximately 500 mm^3^, whereas tumor volumes in the remaining groups remained below this threshold. Although the CMVtt-treated group exhibited the smallest mean tumor volume at endpoint, this difference was not statistically significant by one-way ANOVA.

**Figure 7:**
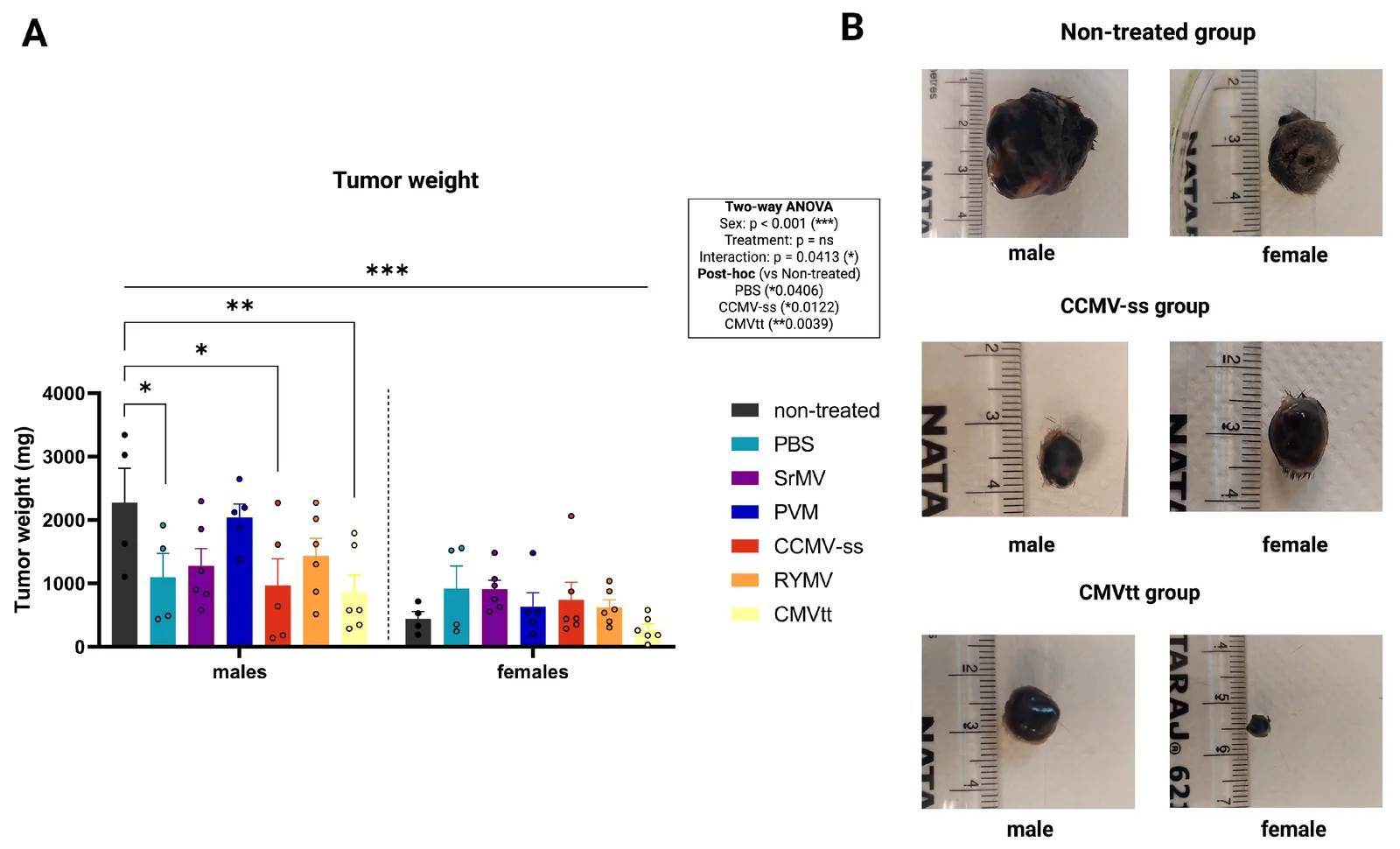
Endpoint tumor burden following intratumoral administration of plant-derived VLPs. (**A**) Endpoint tumor weights in male and female mice from the non-treated, PBS, SrMV, PVM, CCMV-ss, RYMV, and CMVtt groups. Individual data points and mean ± SEM are shown. Two-way ANOVA was used to assess the effects of host sex, treatment, and their interaction. Dunnett’s multiple-comparisons test was used for post hoc comparisons with the corresponding non-treated group. (**B**) Representative images of excised tumors from non-treated (largest tumors in both sexes), CCMV-ss, and CMVtt (smallest tumors in both sexes) treatment groups at the experimental endpoint. * *p* < 0.05, ** *p* < 0.01, *** *p* < 0.001; ns, not significant.

Analysis of final excised tumor weights showed a pattern broadly consistent with tumor volume measurements. Overall, male mice developed significantly heavier tumors than female mice across groups (Figure 7A), with a strong host sex effect (*p* < 0.001) and a significant host sex × treatment interaction (*p* = 0.0413). No significant main effect of treatment was detected by two-way ANOVA. Post hoc analysis versus non-treated controls indicated significantly lower tumor weights in PBS-, CCMV-ss-, and CMVtt-treated male mice (*p* = 0.0406, *p* = 0.0122, and *p* = 0.0039, respectively). Despite variability, the heaviest tumors were observed in non-treated and PVM-treated male mice, approximately 2000 mg, whereas the smallest tumors in males occurred in the CCMV-ss and CMVtt groups, both remaining below 1000 mg (Figure 7A). In female mice, no group reached a mean tumor weight of 1000 mg, consistent with the slower tumor progression observed in females.

Representative images of excised tumors further supported these findings. The largest tumors from non-treated male mice were visibly larger than the largest tumors observed in non-treated female mice (Figure 7B, top row). In males, CCMV-ss treatment was associated with visibly smaller tumors compared with the non-treated condition (Figure 7B, middle row). CMVtt treatment was associated with reduced tumor burden in both sexes, with the smallest tumors observed in the CMVtt-treated groups (Figure 7B, bottom row). These representative images are consistent with the quantitative tumor volume and endpoint tumor weight data, but should be interpreted as illustrative examples rather than independent statistical evidence.

Notably, reduced tumor weight was also observed in PBS-treated males, indicating that nonspecific effects associated with intratumoral injection, local tumor perturbation, vehicle delivery, or inflammatory responses to the injection procedure may contribute to endpoint variability. Therefore, the present in vivo data should be interpreted as candidate-prioritization evidence rather than definitive proof of VLP-specific efficacy beyond the procedural/vehicle control. Nevertheless, CCMV-ss- and CMVtt-treated male mice showed the most consistent pattern across tumor volume and endpoint tumor weight, supporting their prioritization for further evaluation.

Importantly, male recipients represent a sex-matched setting for the male-derived B16-F10 tumor model. Therefore, the candidate-associated reductions observed with CCMV-ss and CMVtt in male mice cannot be explained by female anti-H-Y immune recognition. These findings support prioritization of CCMV-ss and CMVtt within this model, while not establishing an intrinsically sex-specific or procedure-independent VLP-specific therapeutic effect.

### 3.7. Tumor–Host Sex Context Influences Interpretation of Tumor-Infiltrating Immune Cell Populations and Systemic Cytokine Profiles

Tumor responses to immunotherapy are strongly influenced by the immune composition of the TME. Accumulation of defined immune cell populations, particularly CD8^+^ T cells, within tumors has been associated with favorable clinical outcomes in several cancer types, including triple-negative breast cancer [59], ovarian cancer [60], head and neck cancer [61], and melanoma [62,63]. These findings are consistent with the concept of cancer immunoediting, in which immune surveillance can suppress tumor development but may ultimately be overcome during tumor progression. Therefore, profiling tumor-infiltrating immune cells, including tumor-infiltrating lymphocytes (TILs), is essential for understanding tumor biology and therapeutic responses [64,65].

**Figure 8:**
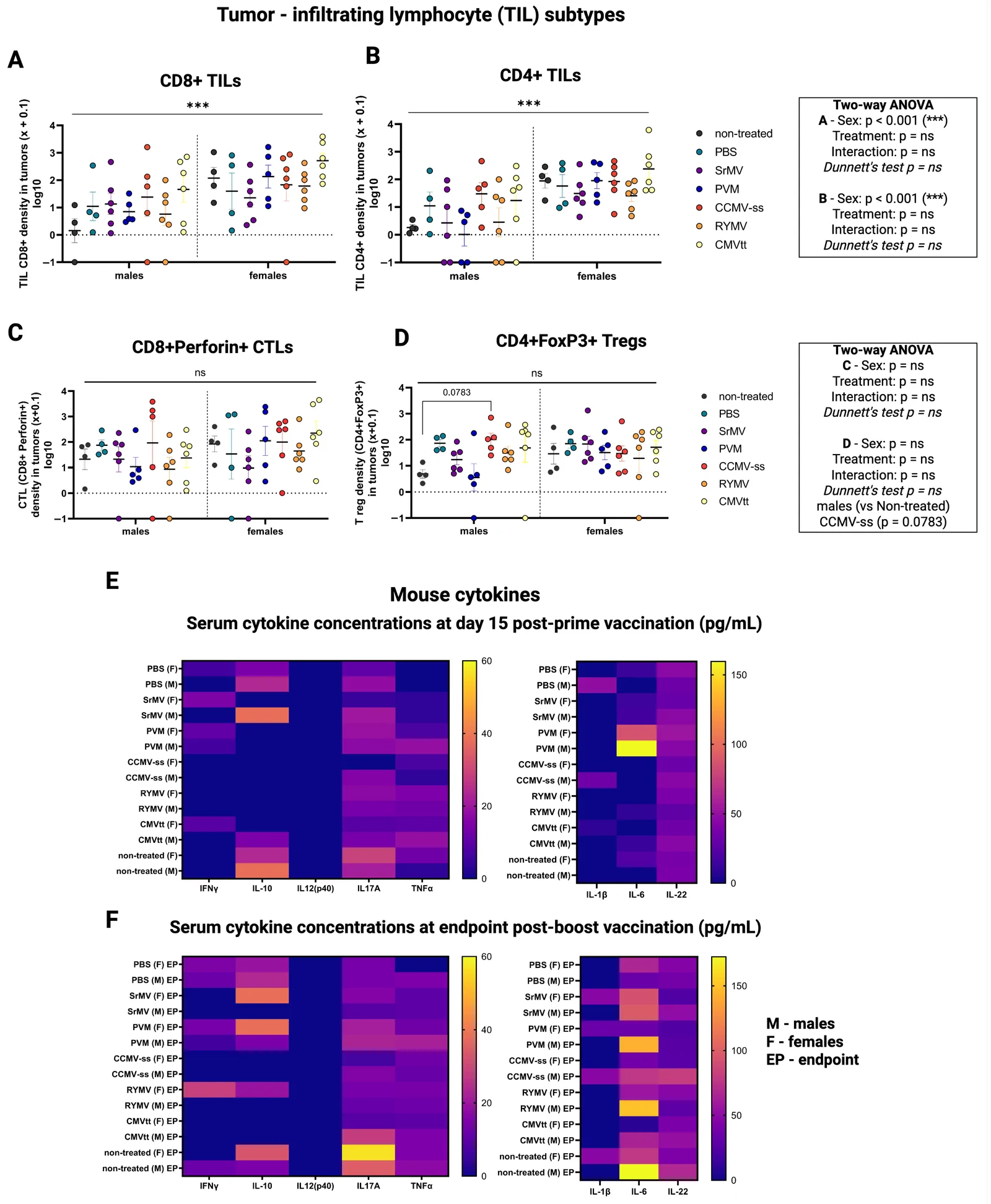
Tumor–host sex context differences and tumor-infiltrating lymphocytes and systemic cytokine responses (**A**–**D**). Quantification of tumor-infiltrating lymphocyte (TIL) subsets in excised tumors from male and female mice. Densities (cell count per tumor mg) of (**A**) CD8^+^ T cells, (**B**) CD4^+^ T cells, (**C**) CD8^+^Perforin^+^ cytotoxic T lymphocytes (CTLs), and (**D**) CD4^+^FoxP3^+^ regulatory T cells (Tregs) are shown as log_10_-transformed TIL densities (x + 0.1). Each dot represents an individual tumor, with horizontal bars indicating group means ± SEM. Two-way ANOVA revealed a significant effect of sex for CD8^+^ and CD4^+^ TILs (*** *p* < 0.001), while no significant treatment or interaction effects were detected. A trend toward reduced Treg density was observed in CCMV-ss–treated male mice compared to non-treated controls ((**D**) *p* = 0.0783, Dunnett’s test). (**E**,**F**) Heatmaps showing serum cytokine concentrations (pg/mL) measured by multiplex analysis at (**E**) day 15 post-prime vaccination and (**F**) experimental endpoint following booster vaccination. Cytokines analyzed include IFN-γ, IL-10, IL-12(p40), IL-17A, TNF-α, IL-1β, IL-6, and IL-22. Data are shown separately for male (M) and female (F) mice. Color intensity indicates cytokine concentration.

Analysis of TILs revealed a significant effect of host sex on CD45.2^+^CD3^+^CD8^+^ T cell density (Figure 8A; *p* < 0.001, two-way ANOVA), with female mice exhibiting higher mean CD8^+^ TIL values across treatment groups compared with males. However, because female recipients represent a tumor–host sex-mismatched setting in the male-derived B16-F10 model, this difference cannot be attributed to host sex alone and may partly reflect immune recognition of male-specific minor histocompatibility antigens, including H-Y antigens. In male recipients, which represent a tumor–host sex-matched setting for this model, CD8^+^ TIL densities were highest in the CMVtt- and CCMV-ss-treated groups and lowest in non-treated controls; however, no significant main treatment or host sex × treatment interaction effect was detected by two-way ANOVA.

A similar pattern was observed for CD45.2^+^CD3^+^CD4^+^ T helper cells (Figure 8B; *p* < 0.001, two-way ANOVA), with female mice showing higher CD4^+^ TIL densities across groups. In males, CCMV-ss- and CMVtt-treated tumors showed higher CD4^+^ TIL values than non-treated controls, whereas no clear treatment-associated differences were observed in females. These findings are consistent with previous reports in murine melanoma models associating higher CD4^+^ and CD8^+^ T cell infiltration in females with reduced tumor growth, while males exhibit lower T cell infiltration and more aggressive tumor progression [58]. In the present study, however, this pattern should be interpreted in the context of the male-derived B16-F10 model, where female recipients may exhibit additional immune activation related to tumor–host sex mismatch.

Analysis of CD8^+^Perforin^+^ cytotoxic T lymphocytes showed no significant effect of host sex and no consistent treatment-associated differences (Figure 8C). Similarly, CD4^+^FoxP3^+^ regulatory T cell densities did not differ significantly between sexes and were not significantly altered by treatment (Figure 8D). A non-significant trend toward altered Treg density was observed in CCMV-ss-treated male mice (*p* = 0.0783). Although elevated Treg infiltration is generally associated with poor clinical outcomes in melanoma [66], our data indicate that tumor control following CCMV-ss vaccination does not result in Treg density reduction. As the CD3^+^CD4^+^FoxP3^+^ gate encompasses both suppressive and activated Treg subsets, the functional significance of this finding cannot be resolved from the present analysis, particularly because the CD3^+^CD4^+^FoxP3^+^ gate may include functionally distinct Treg populations [67].

Tumor-infiltrating myeloid populations were also assessed. CD11b^+^MHCII^+^CD11c^+^ cell density showed a significant effect of host sex (*p* < 0.001, two-way ANOVA), with higher values observed in female mice than in males. In male mice, CCMV-ss and CMVtt treatment was associated with increased CD11b^+^MHCII^+^CD11c^+^ cell density compared with non-treated controls (*p* = 0.0136 and *p* = 0.0074, respectively; Supplementary Figure S6B). CD11b^+^MHCII^+^CD11c^−^ cell densities were also higher in females than in males (*p* = 0.0273, two-way ANOVA; Supplementary Figure S6A), with no significant treatment-associated changes detected.

Taken together, these findings show that tumor–host sex context was associated with differences in tumor immune composition in the B16-F10 melanoma model. Female recipients displayed higher infiltration of both lymphoid and myeloid populations across treatment groups, but this pattern may reflect tumor–host sex mismatch and H-Y antigen-mediated immune recognition in addition to intrinsic host sex-associated biology. In male recipients, which represent a tumor–host sex-matched setting for the male-derived B16-F10 model, CCMV-ss and CMVtt VLP treatment was associated with increased infiltration of T cells and CD11b^+^MHCII^+^CD11c^+^ myeloid cells relative to non-treated controls, corresponding to the groups with the strongest reductions in tumor burden.

Systemic cytokine levels were analyzed in serum collected after the first intratumoral treatment, day 15, and at endpoint after the booster treatment (Figure 8E,F). IFN-γ and IL-12(p40) levels remained low across groups at both time points. Distinct cytokine patterns were observed between treatment groups and between sexes, with IL-17A showing group-dependent variation. Female mice generally exhibited lower IL-6 levels across groups, whereas elevated IL-6 was observed in non-treated and PVM-treated male mice, which also displayed higher tumor weights (Figure 8E,F). This is consistent with the established role of IL-6 in promoting tumor progression [68]. In contrast, the most effective male treatment groups, particularly CCMV-ss and CMVtt, did not show elevated systemic IL-6, IL-10, or TNF-α levels.

Overall, local immune profiling and systemic cytokine analysis support an association between VLP-induced immune remodeling and antitumor activity in male B16-F10 tumor-bearing mice. Because male recipients represent the tumor–host sex-matched setting for the male-derived B16-F10 model, the immune changes observed in CCMV-ss- and CMVtt-treated males are not explained by female anti-H-Y immune recognition. In contrast, comparisons between male and female recipients should be interpreted with caution because female recipients may exhibit additional immune activation driven by tumor–host sex mismatch.

## 4. Conclusions

In this study, we systematically evaluated a panel of plant virus-derived VLPs as candidates for intratumoral cancer immunotherapy and established an integrated comparative framework combining structural characterization, functional immune assays, innate immune reporter analysis, and in vivo validation. Using an endotoxin-minimized ClearColi^TM^ BL21 (DE3) production system, we produced eleven plant VLPs representing both filamentous and icosahedral architectures. The resulting VLPs differed in assembly efficiency, RNA encapsidation, production yield, macrophage activation, tumor cell killing activity, TLR3 engagement, and in vivo antitumor performance. Notably, four VLPs, SrMV, PVX, BBMV, and ApMV, are described here for the first time in an *E. coli* expression system. Although ClearColi-based production resulted in lower yields than conventional strains, the reduced endotoxin background enabled improved assessment of VLP-associated immunostimulatory activity.

ClearColi-expressed coat proteins were soluble and consistently assembled into VLPs resembling their native viral structures after purification. Production yields were lower and culture growth was slower than typically observed with conventional BL21 (DE3) [6,23] or C2566 *E. coli* strains [7,8,9,10,11,22], a limitation previously attributed to the multiple genetic modifications introduced into ClearColi to reduce endotoxin activity [69,70]. These modifications result in synthesis of detoxified lipid IV_a_ instead of hexa-acylated lipid A, leading to markedly reduced TLR4-mediated immunostimulation [71]. Endotoxin levels in all VLP preparations were maintained below 100 EU/mg protein, ensuring that the final concentration in vitro did not exceed 1 EU/mL at the highest dose and that in vivo endotoxin exposure remained ≤2.5 EU or ≤5 EU per injection at 25 µg and 50 µg dosing, respectively. Consequently, residual endotoxin in ClearColi-derived preparations was unlikely to be a major source of nonspecific immune stimulation relative to conventional *E. coli*-based production systems.

Preliminary in vitro analyses suggest that several VLPs activated macrophages and promoted tumor cell killing. However, classical M1/M2 polarization markers did not consistently predict cytotoxic activity, indicating that marker-based macrophage classification alone was insufficient to capture functionally relevant antitumor responses. SrMV VLPs showed the strongest M1-associated macrophage activation profile, whereas PVM and CCMV-ss VLPs induced moderate activation and CMVtt VLPs showed comparatively limited effects by these markers. Despite this, CMVtt most effectively enhanced macrophage-associated tumor cell killing under limiting effector-to-target conditions, highlighting the importance of functional immune assays alongside phenotypic characterization.

HEK-Blue^TM^ hTLR3 reporter assays provided an exploratory comparative assessment of TLR3-associated activity among selected intact VLP preparations. CMVtt and RYMV induced the strongest reporter responses, whereas SrMV, CCMV-ss, and PVM showed limited activity under RNA-normalized conditions. Reporter activation was not predicted solely by total RNA content or apparent RNA size distribution, and no direct relationship was observed between the nucleic acid signals detected by native agarose gel electrophoresis in Figure 1C and immunostimulatory activity across the broader VLP panel. Although these findings are consistent with established TLR3 biology, in which RNA length, secondary structure, and accessibility can influence endosomal receptor activation [14], the present experiments do not identify the molecular features responsible for the observed differences. Because intact VLP preparations were tested, possible contributions from RNA sequence or secondary structure, RNA accessibility, particle uptake and endosomal processing, capsid-associated properties, or other preparation-associated components cannot be distinguished by the current experimental design. Moreover, the weak TLR3 reporter activity of CCMV-ss despite its favorable in vivo profile indicates that TLR3 signaling is not an independent or universal predictor of therapeutic performance and that complementary innate sensing pathways may contribute to VLP-mediated immune activity. Future studies using nuclease-sensitivity assays, RNA-depleted or disrupted particles, targeted RNA sequence and structural analyses, and complementary innate immune reporter systems will be required to define the relevant immunostimulatory features and sensing pathways. Collectively, these findings demonstrate that the immunostimulatory activity of plant-derived VLPs likely reflects an interplay between RNA- and particle-associated properties and support the use of multiple complementary functional assays rather than reliance on a single innate immune reporter system for candidate selection.

In vivo candidate performance was evaluated in the poorly immunogenic, male-derived B16-F10 melanoma model. Within this model, tumor–host sex context was associated with differences in tumor progression, baseline immune infiltration, and treatment outcome. Female mice showed slower tumor growth, higher infiltration of CD4^+^ and CD8^+^ T cells and antigen-presenting myeloid populations, and lower variability between treatment groups. In contrast, the most consistent candidate-associated effects were observed primarily in male mice. In males, CCMV-ss and CMVtt treatment groups showed reduced tumor progression trends and increased T cell and CD11b^+^MHCII^+^CD11c^+^ myeloid cell infiltration, supporting their prioritization as lead candidates for further evaluation.

The observed antitumor efficacy of CCMV-ss and CMVtt VLPs—including delayed tumor progression and increased immune cell infiltration in males—highlights their potential as immunotherapy candidates. However, interpretation of these in vivo findings requires consideration of two key experimental factors. First, the use of male-derived B16-F10 melanoma cells in female hosts introduces the potential for H-Y antigen-driven immunogenicity, which may confound host sex-associated comparisons [53]. Consequently, our findings should not be interpreted as definitive evidence of intrinsic sex-dependent therapeutic mechanisms. Future studies using sex-matched syngeneic models will be essential to define the broader relevance of these observations. Second, the nonspecific tumor weight reduction noted in PBS-injected controls indicates that intratumoral injection itself can trigger a measurable procedural or inflammatory effect. Consequently, the observed tumor and immune responses may reflect the combined contribution of VLP-mediated stimulation, vehicle injection, and mechanical tumor perturbation. Future experiments should incorporate additional procedural controls, such as sham puncture controls, and should be powered to distinguish VLP-specific activity from procedural and vehicle-associated effects.

The enhanced activity of CMVtt VLPs may relate to incorporation of the tetanus toxoid-derived CD4^+^ T cell helper epitope P2, which has been reported to support T cell help and improve immune responses in C57BL/6, H-2ᵇ, mice [7,72]. The CCMV-ss modification, designed to improve capsid stability [8,21], may likewise contribute to its favorable immunostimulatory performance. Although these proposed explanations remain indirect in the present study, they are consistent with the observed functional activity of both VLPs.

Overall, the in vivo findings only partially matched in vitro macrophage marker profiles, as illustrated by the strong in vitro M1-associated activation profile but limited in vivo efficacy of SrMV VLPs. In contrast, therapeutic performance aligned more closely with functional tumor cell killing and TLR3 activation than with classical macrophage polarization markers alone. This integrated screening approach prioritized CCMV-ss and CMVtt VLPs as the most promising candidates in the panel, based on convergent trends across in vitro immune-functional assays, tumor progression measurements, and local immune infiltration in male B16-F10 melanoma-bearing mice. Collectively, these findings support plant-derived VLPs as versatile immunomodulatory biomaterials for intratumoral cancer immunotherapy and provide a rationale for further optimization of CCMV-ss and CMVtt as lead candidates. To our knowledge, this is the first side-by-side comparison of structurally diverse plant virus-derived VLPs produced under endotoxin-minimized conditions and evaluated across macrophage activation, tumor cell killing, RNA-dependent innate immune signaling, and intratumoral antitumor activity.

## Figures and Tables

**Table 1:** Comparative summary of ClearColi-expressed plant-derived VLP preparations. Approximate production yields, particle morphology, and detectable nucleic acid signals are summarized from SDS-PAGE, TEM, and NAG analyses. Lane numbers correspond to the SDS-PAGE and NAG analyses shown in Figure 1A and Figure 1C, respectively. NAG, native agarose gel electrophoresis.

VLP	Figure 1A/C Lane	Structural Class/ Family	Approximate Yield	TEM Morphology	NAG Signal	Key Characterization Outcome
**EMV**	1	Icosahedral/ *Tymoviridae*	~15 mg/L	~30 nm particles; heterogeneous, with partially assembled structures	Detected	High yield, but heterogeneous assembly
**SrMV**	2	Icosahedral/ *Tymoviridae*	~4 mg/L	~30 nm particles; relatively homogeneous	Detected	Newly produced in *E. coli*; homogeneous particle population
**BBMV**	3	Icosahedral/ *Bromoviridae*	~9.5 mg/L	Predominantly T = 3 particles, ~30–40 nm	Detected	Newly produced in *E. coli*; good assembly profile
**ApMV**	4	Icosahedral/ *Bromoviridae*	~2.4 mg/L	Pleomorphic particles and aggregates	Detected	Heterogeneous assembly
**PVY**	5	Filamentous/ *Potyviridae*	~6.7 mg/L	Flexible filaments, ~600–1000 nm	Not detected	Filamentous particles with nucleic acid below the detection threshold of NAG
**PVM**	6	Filamentous/ *Betaflexiviridae*	~1 mg/L	Short rigid filaments, ~200 nm; some aggregates	Detected	Short filamentous assemblies with detectable nucleic acid
**PVX**	7	Filamentous/ *Alphaflexiviridae*	~0.3 mg/L	Short heterogeneous filaments, ~40–200 nm	Not detected	Newly produced in *E. coli*; low yield and heterogeneous assembly
**CCMV-ss**	8	Icosahedral/ *Bromoviridae*	~10 mg/L	Predominantly T = 3 particles, ~30–40 nm	Detected	Stable, well-assembled particles
**CfMV**	9	Icosahedral/ *Solemoviridae*	~2.5 mg/L	~40 nm particles with fragments and aggregates	Detected	Reduced assembly quality in ClearColi
**RYMV**	10	Icosahedral/ *Solemoviridae*	~1.7 mg/L	~40 nm particles with fragments and pleomorphic structures	Detected	Heterogeneous particle population with detectable nucleic acid
**CMVtt**	11	Icosahedral/ *Bromoviridae*	~7 mg/L	Predominantly T = 3 particles, ~30–40 nm	Detected; strongest signal	Well-assembled particles with the strongest NAG signal

## Data Availability

Data are available upon reasonable request. All data are available in the main text or the Supplementary Materials or upon reasonable request from the corresponding author.

## Supplementary Materials

The following supporting information can be downloaded at: https://www.mdpi.com/article/10.3390/vaccines14080697/s1. Figure S1: Description of plasmid pET28-ApMV. Figure S2: Dynamic light scattering (DLS) analysis of coat proteins (CPs) from plant viruses. Figure S3: Dynamic light scattering (DLS) analysis of coat proteins (CPs) from plant viruses. Figure S4: Dynamic light scattering (DLS) analysis of coat proteins (CPs) from plant viruses. Figure S5: Dynamic light scattering (DLS) analysis of coat proteins (CPs) from plant viruses. Figure S6: Representative unstained RAW264.7 macrophage controls used to assess background fluorescence in flow-cytometry analysis. Figure S7: Quantification of tumor-infiltrating myeloid cell populations in excised tumors from male and female mice. Table S1: Condition-matched B16-F10-luc2 stimulus-only controls used for co-culture normalization.

## References

[1] Zhang Y., Zhang Z. (2020). The history and advances in cancer immunotherapy: Understanding the characteristics of tumor-infiltrating immune cells and their therapeutic implications. Cell. Mol. Immunol..

[2] Jiang Z., Fu Y., Shen H. (2024). Development of Intratumoral Drug Delivery Based Strategies for Antitumor Therapy. Drug Des. Dev. Ther..

[3] Yun W.S., Kim J., Lim D.-K., Kim D.-H., Jeon S.I., Kim K. (2023). Recent Studies and Progress in the Intratumoral Administration of Nano-Sized Drug Delivery Systems. Nanomaterials.

[4] Lizotte P.H., Wen A.M., Sheen M.R., Fields J., Rojanasopondist P., Steinmetz N.F., Fiering S. (2016). In situ vaccination with cowpea mosaic virus nanoparticles suppresses metastatic cancer. Nat. Nanotechnol..

[5] Pan Y., Yu Y., Wang X., Zhang T. (2020). Tumor-Associated Macrophages in Tumor Immunity. Front. Immunol..

[6] Kalnciema I., Balke I., Skrastina D., Ose V., Zeltins A. (2015). Potato Virus M-Like Nanoparticles: Construction and Characterization. Mol. Biotechnol..

[7] Zeltins A., West J., Zabel F., El Turabi A., Balke I., Haas S., Maudrich M., Storni F., Engeroff P., Jennings G.T. (2017). Incorporation of tetanus-epitope into virus-like particles achieves vaccine responses even in older recipients in models of psoriasis, Alzheimer’s and cat allergy. npj Vaccines.

[8] Zinkhan S., Ogrina A., Balke I., Reseviča G., Zeltins A., de Brot S., Lipp C., Chang X., Zha L., Vogel M. (2021). The impact of size on particle drainage dynamics and antibody response. J. Control. Release.

[9] Ogrina A., Skrastina D., Balke I., Kalnciema I., Jansons J., Bachmann M.F., Zeltins A. (2022). Comparison of Bacterial Expression Systems Based on Potato Virus Y-like Particles for Vaccine Generation. Vaccines.

[10] Ogrina A., Balke I., Kalnciema I., Skrastina D., Jansons J., Bachmann M.F., Zeltins A. (2023). Bacterial expression systems based on Tymovirus-like particles for the presentation of vaccine antigens. Front. Microbiol..

[11] Sobczak J.M., Barkovska I., Balke I., Rothen D.A., Mohsen M.O., Skrastina D., Ogrina A., Martina B., Jansons J., Bogans J. (2024). Identifying Key Drivers of Efficient B Cell Responses: On the Role of T Help, Antigen-Organization, and Toll-like Receptor Stimulation for Generating a Neutralizing Anti-Dengue Virus Response. Vaccines.

[12] Zeltins A. (2013). Construction and characterization of virus-like particles: A review. Mol. Biotechnol..

[13] Bachmann M.F., Jennings G.T. (2010). Vaccine delivery: A matter of size, geometry, kinetics and molecular patterns. Nat. Rev. Immunol..

[14] Kawai T., Akira S. (2010). The role of pattern-recognition receptors in innate immunity: Update on Toll-like receptors. Nat. Immunol..

[15] Mohsen M.O., Bachmann M.F. (2022). Virus-like particle vaccinology, from bench to bedside. Cell. Mol. Immunol..

[16] Mao C., Beiss V., Fields J., Steinmetz N.F., Fiering S. (2021). Cowpea mosaic virus stimulates antitumor immunity through recognition by multiple MYD88-dependent toll-like receptors. Biomaterials.

[17] Wang C., Fiering S.N., Steinmetz N.F. (2019). Cowpea Mosaic Virus Promotes Anti-Tumor Activity and Immune Memory in a Mouse Ovarian Tumor Model. Adv. Ther..

[18] de Oliveira J.F.A., Moreno-Gonzalez M.A., Ma Y., Deng X., Schuphan J., Steinmetz N.F. (2024). Plant Virus Intratumoral Immunotherapy with CPMV and PVX Elicits Durable Antitumor Immunity in a Mouse Model of Diffuse Large B-Cell Lymphoma. Mol. Pharm..

[19] Shukla S., Wang C., Beiss V., Cai H., Washington T., Murray A.A., Gong X., Zhao Z., Masarapu H., Zlotnick A. (2020). The unique potency of Cowpea mosaic virus (CPMV) *in situ* cancer vaccine. Biomater. Sci..

[20] Conforti F., Pala L., Bagnardi V., De Pas T., Martinetti M., Viale G., Gelber R.D., Goldhirsch A. (2018). Cancer immunotherapy efficacy and patients’ sex: A systematic review and meta-analysis. Lancet Oncol..

[21] Bancroft J.B., Rees M.W., Johnson M.W., Dawson J.R.O. (1973). A salt-stable mutant of cowpea chlorotic mottle virus. J. Gen. Virol..

[22] Kalnciema I., Skrastina D., Ose V., Pumpens P., Zeltins A. (2012). Potato virus Y-like particles as a new carrier for the presentation of foreign protein stretches. Mol. Biotechnol..

[23] Balke I., Reseviča G., Zeltins A. (2018). Isolation and Characterization of Two Distinct Types of Unmodified Spherical Plant Sobemovirus-Like Particles for Diagnostic and Technical Uses. Methods Mol. Biol..

[24] Masarapu H., Patel B.K., Chariou P.L., Hu H., Gulati N.M., Carpenter B.L., Ghiladi R.A., Shukla S., Steinmetz N.F. (2017). Physalis Mottle Virus-Like Particles as Nanocarriers for Imaging Reagents and Drugs. Biomacromolecules.

[25] Schwarz B., Morabito K.M., Ruckwardt T.J., Patterson D.P., Avera J., Miettinen H.M., Graham B.S., Douglas T. (2016). Viruslike Particles Encapsidating Respiratory Syncytial Virus M and M2 Proteins Induce Robust T Cell Responses. ACS Biomater. Sci. Eng..

[26] Gibbs A., Mackenzie A.M. (1998). Tymovirus isolation and genomic RNA extraction. Methods Mol. Biol..

[27] Lavelle L., Gingery M., Phillips M., Gelbart W.M., Knobler C.M., Cadena-Nava R.D., Vega-Acosta J.R., Pinedo-Torres L.A., Ruiz-Garcia J. (2009). Phase diagram of self-assembled viral capsid protein polymorphs. J. Phys. Chem. B.

[28] Arkhipenko M.V., Petrova E.K., Nikitin N.A., Protopopova A.D., Dubrovin E.V., Yaminskii I.V., Rodionova N.P., Karpova O.V., Atabekov J.G. (2011). Characteristics of Artificial Virus-like Particles Assembled in vitro from Potato Virus X Coat Protein and Foreign Viral RNAs. Acta Naturae.

[29] Qu C., Liljas L., Opalka N., Brugidou C., Yeager M., Beachy R.N., Fauquet C.M., Johnson J.E., Lin T. (2000). 3D domain swapping modulates the stability of members of an icosahedral virus group. Structure.

[30] Bessa J., Jegerlehner A., Hinton H.J., Pumpens P., Saudan P., Schneider P., Bachmann M.F. (2009). Alveolar macrophages and lung dendritic cells sense RNA and drive mucosal IgA responses. J. Immunol..

[31] Zabel F., Fettelschoss A., Vogel M., Johansen P., Kündig T.M., Bachmann M.F. (2017). Distinct T helper cell dependence of memory B-cell proliferation versus plasma cell differentiation. Immunology.

[32] Wang C., Beiss V., Steinmetz N.F. (2019). Cowpea Mosaic Virus Nanoparticles and Empty Virus-Like Particles Show Distinct but Overlapping Immunostimulatory Properties. J. Virol..

[33] Lučiūnaitė A., Dalgėdienė I., Žilionis R., Mašalaitė K., Norkienė M., Šinkūnas A., Gedvilaitė A., Kučinskaitė-Kodzė I., Žvirblienė A. (2022). Activation of NLRP3 Inflammasome by Virus-Like Particles of Human Polyomaviruses in Macrophages. Front. Immunol..

[34] Mantovani A., Marchesi F., Malesci A., Laghi L., Allavena P. (2017). Tumour-associated macrophages as treatment targets in oncology. Nat. Rev. Clin. Oncol..

[35] Kröger M., Scheffel J., Shirshin E.A., Schleusener J., Meinke M.C., Lademann J., Maurer M., E Darvin M. (2022). Label-free imaging of M1 and M2 macrophage phenotypes in the human dermis in vivo using two-photon excited FLIM. eLife.

[36] Korotkaja K., Jansons J., Spunde K., Rudevica Z., Zajakina A. (2023). Establishment and Characterization of Free-Floating 3D Macrophage Programming Model in the Presence of Cancer Cell Spheroids. Int. J. Mol. Sci..

[37] Dorrington M.G., Fraser I.D.C. (2019). NF-κB Signaling in Macrophages: Dynamics, Crosstalk, and Signal Integration. Front. Immunol..

[38] Mills C.D., Kincaid K., Alt J.M., Heilman M.J., Hill A.M. (2000). M-1/M-2 macrophages and the Th1/Th2 paradigm. J. Immunol..

[39] Murray P.J., Allen J.E., Biswas S.K., Fisher E.A., Gilroy D.W., Goerdt S., Gordon S., Hamilton J.A., Ivashkiv L.B., Lawrence T. (2014). Macrophage activation and polarization: Nomenclature and experimental guidelines. Immunity.

[40] Noy R., Pollard J.W. (2014). Tumor-associated macrophages: From mechanisms to therapy. Immunity.

[41] Movahedi K., Laoui D., Gysemans C., Baeten M., Stangé G., Van den Bossche J., Mack M., Pipeleers D., Veld P.I., De Baetselier P. (2010). Different tumor microenvironments contain functionally distinct subsets of macrophages derived from Ly6C(high) monocytes. Cancer Res..

[42] Antonsen K.W., Jensen A.G., Carstensen M., Nejsum L.N., Sorensen B.S., Etzerodt A., Moestrup S.K., Møller H.J. (2024). Proinflammatory polarization strongly reduces human macrophage in vitro phagocytosis of tumor cells in response to CD47 blockade. Eur. J. Immunol..

[43] Tedesco S., Scattolini V., Albiero M., Bortolozzi M., Avogaro A., Cignarella A., Fadini G.P. (2019). Mitochondrial Calcium Uptake Is Instrumental to Alternative Macrophage Polarization and Phagocytic Activity. Int. J. Mol. Sci..

[44] Andreu P., Johansson M., Affara N.I., Pucci F., Tan T., Junankar S., Korets L., Lam J., Tawfik D., DeNardo D.G. (2010). FcRgamma activation regulates inflammation-associated squamous carcinogenesis. Cancer Cell.

[45] DeNardo D.G., Barreto J.B., Andreu P., Vasquez L., Tawfik D., Kolhatkar N., Coussens L.M. (2009). CD4(+) T cells regulate pulmonary metastasis of mammary carcinomas by enhancing protumor properties of macrophages. Cancer Cell.

[46] Ruffell B., Coussens L.M. (2015). Macrophages and therapeutic resistance in cancer. Cancer Cell.

[47] Takeuchi O., Akira S. (2010). Pattern recognition receptors and inflammation. Cell.

[48] Zepeda-Cervantes J., Ramírez-Jarquín J.O., Vaca L. (2020). Interaction Between Virus-Like Particles (VLPs) and Pattern Recognition Receptors (PRRs) From Dendritic Cells (DCs): Toward Better Engineering of VLPs. Front. Immunol..

[49] Chang X., Krenger P., Krueger C.C., Zha L., Han J., Yermanos A., Roongta S., Mohsen M.O., Oxenius A., Vogel M. (2022). TLR7 Signaling Shapes and Maintains Antibody Diversity Upon Virus-Like Particle Immunization. Front. Immunol..

[50] Krenger P.S., Josi R., Sobczak J., Velazquez T.L.C., Balke I., Skinner M.A., Kramer M.F., Scott C.J.W., Hewings S., Heath M.D. (2024). Influence of antigen density and TLR ligands on preclinical efficacy of a VLP-based vaccine against peanut allergy. Allergy.

[51] Zhang C., Sui Y., Liu S., Yang M. (2024). In vitro and in vivo experimental models for cancer immunotherapy study. Curr. Res. Biotechnol..

[52] Scott D.M., Ehrmann I.E., Ellis P.S., Bishop C.E., Agulnik A.I., Simpson E., Mitchell M.J. (1995). Identification of a mouse male-specific transplantation antigen, H-Y. Nature.

[53] Conforti C., Zalaudek I. (2021). Epidemiology and Risk Factors of Melanoma: A Review. Dermatol. Pract. Concept..

[54] Markman J.L., Porritt R.A., Wakita D., Lane M.E., Martinon D., Rivas M.N., Luu M., Posadas E.M., Crother T.R., Arditi M. (2020). Loss of testosterone impairs anti-tumor neutrophil function. Nat. Commun..

[55] Mohsen M.O., Vogel M., Riether C., Muller J., Salatino S., Ternette N., Gomes A.C., Cabral-Miranda G., El-Turabi A., Ruedl C. (2019). Targeting Mutated Plus Germline Epitopes Confers Pre-clinical Efficacy of an Instantly Formulated Cancer Nano-Vaccine. Front. Immunol..

[56] Zhang Y., Lin Z., Wan Y., Cai H., Deng L., Li R. (2019). The Immunogenicity and Anti-tumor Efficacy of a Rationally Designed Neoantigen Vaccine for B16F10 Mouse Melanoma. Front. Immunol..

[57] Mantilla-Rojas C., Velasquez F.C., Morton J.E., Clemente L.C., Parra E.R., Torres-Cabala C., Sevick-Muraca E.M. (2022). Enhanced T-Cell Priming and Improved Anti-Tumor Immunity through Lymphatic Delivery of Checkpoint Blockade Immunotherapy. Cancers.

[58] Dakup P.P., Porter K.I., Little A.A., Zhang H., Gaddameedhi S. (2020). Sex differences in the association between tumor growth and T cell response in a melanoma mouse model. Cancer Immunol. Immunother..

[59] Rudqvist N.-P., Charpentier M., Lhuillier C., Wennerberg E., Spada S., Sheridan C., Zhou X.K., Zhang T., Formenti S.C., Sims J.S. (2023). Immunotherapy targeting different immune compartments in combination with radiation therapy induces regression of resistant tumors. Nat. Commun..

[60] Zhang L., Conejo-Garcia J.R., Katsaros D., Gimotty P.A., Massobrio M., Regnani G., Makrigiannakis A., Gray H., Schlienger K., Liebman M.N. (2003). Intratumoral T cells, recurrence, and survival in epithelial ovarian cancer. N. Engl. J. Med..

[61] Josi R., Speiser D.E., de Brot S., Vogt A.-C., Sevick-Muraca E.M., Tolstonog G.V., Bachmann M.F., Mohsen M.O. (2024). A tetravalent nanovaccine that inhibits growth of HPV-associated head and neck carcinoma via dendritic and T cell activation. iScience.

[62] Mohsen M.O., Heath M.D., Cabral-Miranda G., Lipp C., Zeltins A., Sande M., Stein J.V., Riether C., Roesti E., Zha L. (2019). Vaccination with nanoparticles combined with micro-adjuvants protects against cancer. J. Immunother. Cancer.

[63] Garduño R.C., Spitschak A., Pannek T., Pützer B.M. (2025). CD8+ T Cell Subsets as Biomarkers for Predicting Checkpoint Therapy Outcomes in Cancer Immunotherapy. Biomedicines.

[64] Maibach F., Sadozai H., Jafari S.M.S., Hunger R.E., Schenk M. (2020). Tumor-Infiltrating Lymphocytes and Their Prognostic Value in Cutaneous Melanoma. Front. Immunol..

[65] Vargas G.M., Shafique N., Xu X., Karakousis G. (2024). Tumor-infiltrating lymphocytes as a prognostic and predictive factor for Melanoma. Expert Rev. Mol. Diagn..

[66] Huang L., Guo Y., Liu S., Wang H., Zhu J., Ou L., Xu X. (2021). Targeting regulatory T cells for immunotherapy in melanoma. Mol. Biomed..

[67] Attias M., Alvarez F., Al-Aubodah T.-A., Istomine R., McCallum P., Huang F., Sleiman A., Nishimura T., del Rincón S.V., Riazalhosseini Y. (2025). Anti-PD-1 amplifies costimulation in melanoma-infiltrating Th1-like Foxp3+ regulatory T cells to alleviate local immunosuppression. J. Immunother. Cancer.

[68] Wu S., Cao Z., Lu R., Zhang Z., Sethi G., You Y. (2025). Interleukin-6 (IL-6)-associated tumor microenvironment remodelling and cancer immunotherapy. Cytokine Growth Factor Rev..

[69] Hunt J.P., Zhao E.L., Soltani M., Frei M., Nelson J.A.D., Bundy B.C. (2019). Streamlining the preparation of “endotoxin-free” ClearColi cell extract with autoinduction media for cell-free protein synthesis of the therapeutic protein crisantaspase. Synth. Syst. Biotechnol..

[70] Cardoso V.M., Paredes S.A.H., Campani G., Gonçalves V.M., Zangirolami T.C. (2022). ClearColi as a platform for untagged pneumococcal surface protein A production: Cultivation strategy, bioreactor culture, and purification. Appl. Microbiol. Biotechnol..

[71] Ueda T., Akuta T., Kikuchi-Ueda T., Imaizumi K., Ono Y. (2016). Improving the soluble expression and purification of recombinant human stem cell factor (SCF) in endotoxin-free Escherichia coli by disulfide shuffling with persulfide. Protein Expr. Purif..

[72] Ahlborg N., Ling I.T., Holder A.A., Riley E.M. (2000). Linkage of exogenous T-cell epitopes to the 19-kilodalton region of Plasmodium yoelii merozoite surface protein 1 (MSP1(19)) can enhance protective immunity against malaria and modulate the immunoglobulin subclass response to MSP1(19). Infect. Immun..

