# Supplementary material for: Comparative Evaluation of Plant-Derived Virus-like Particles as Intratumoral Immunotherapy Agents
Source: Vaccines (Basel). 2026 Aug 12;14(8):697. doi: 10.3390/vaccines14080697 (PMC13517190; doi:10.3390/vaccines14080697)
Supplement: Supplementary file 1 [file vaccines-14-00697-s001.zip › vaccines-4415491-supplementary.pdf]

Supplementary Figure S1

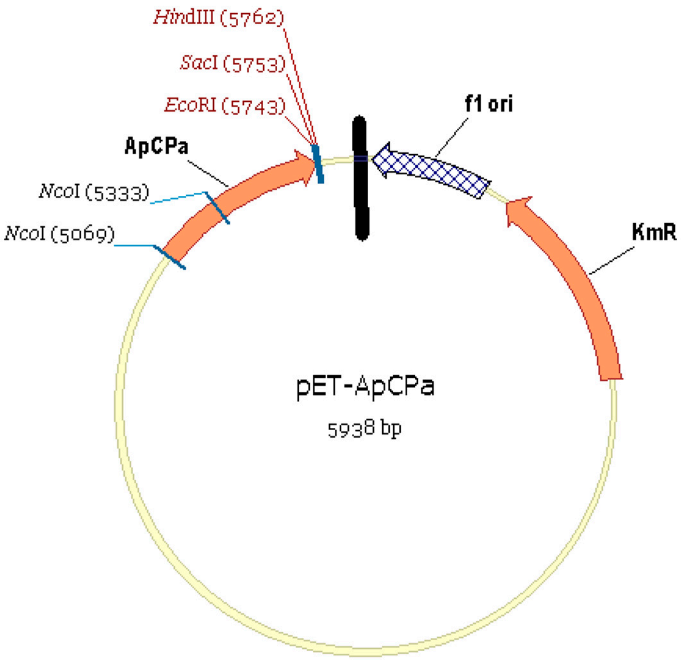

Coat protein (CP) sequence for Apple mosaic virus (ApMV):
MVCKYCNHTHPGSCAGCKWCHGTNRPAPPKRAVARAQANPNKGKNSVRGYPVKEGPSVRRTAWEVRGPNVEPKIPRGHR
VLSSREVTAMVEGKFVNIDFADVFRDLLEKDLKVYTFIIRVNSLSSNGWIGLVEDYDESNPKGPNPMDRKGFKKDQPRGWQ
WEAPPNTTFDDFVRKFRLVLEFKTNFAAGAKVFMRDLYMITSELPPVQIPTNVLLIDEDLLEL*EF

Suppl. Fig. S1. Description of plasmid pET28-ApMV. The expression vector ensures the synthesis of ApMV CP. A plasmid map with single-cut restriction enzyme sites and amino acid sequence of ApMV CP.

Supplementary Figure S2

A

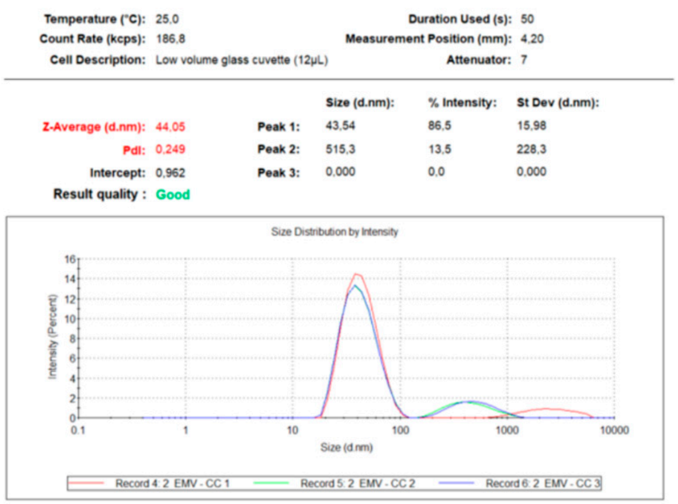

B

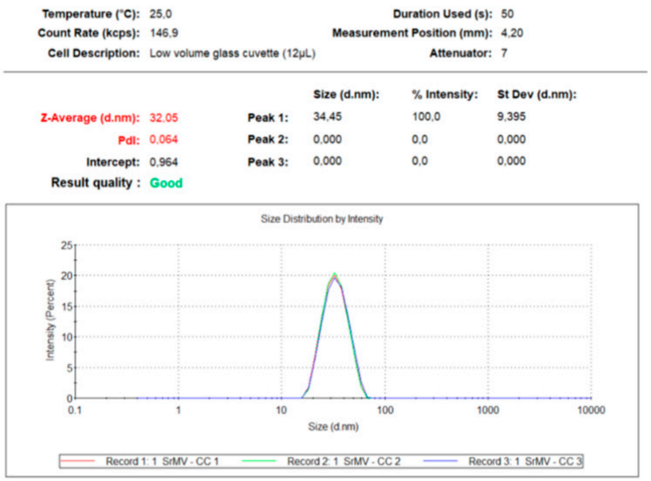

C

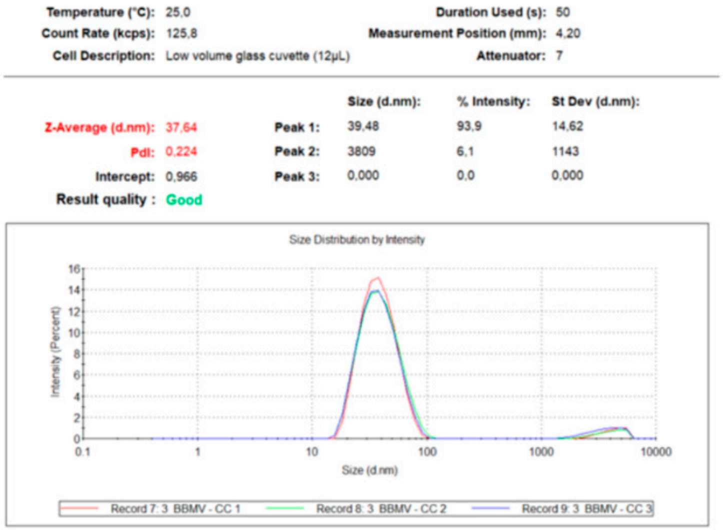

Suppl. Fig.S2. Dynamic light scattering (DLS) analysis of coat proteins (CPs) from plant viruses. Size distribution profiles (intensity-weighted) are shown for (**A**) EMV CP, (**B**) SrMV CP, and (**C**) BBMV CP. Measurements were performed at 25 °C using low-volume glass cuvettes. Z-average diameter and polydispersity index (PdI) values are indicated for each sample. Curves represent three technical replicates per protein preparation.

Supplementary Figure S3

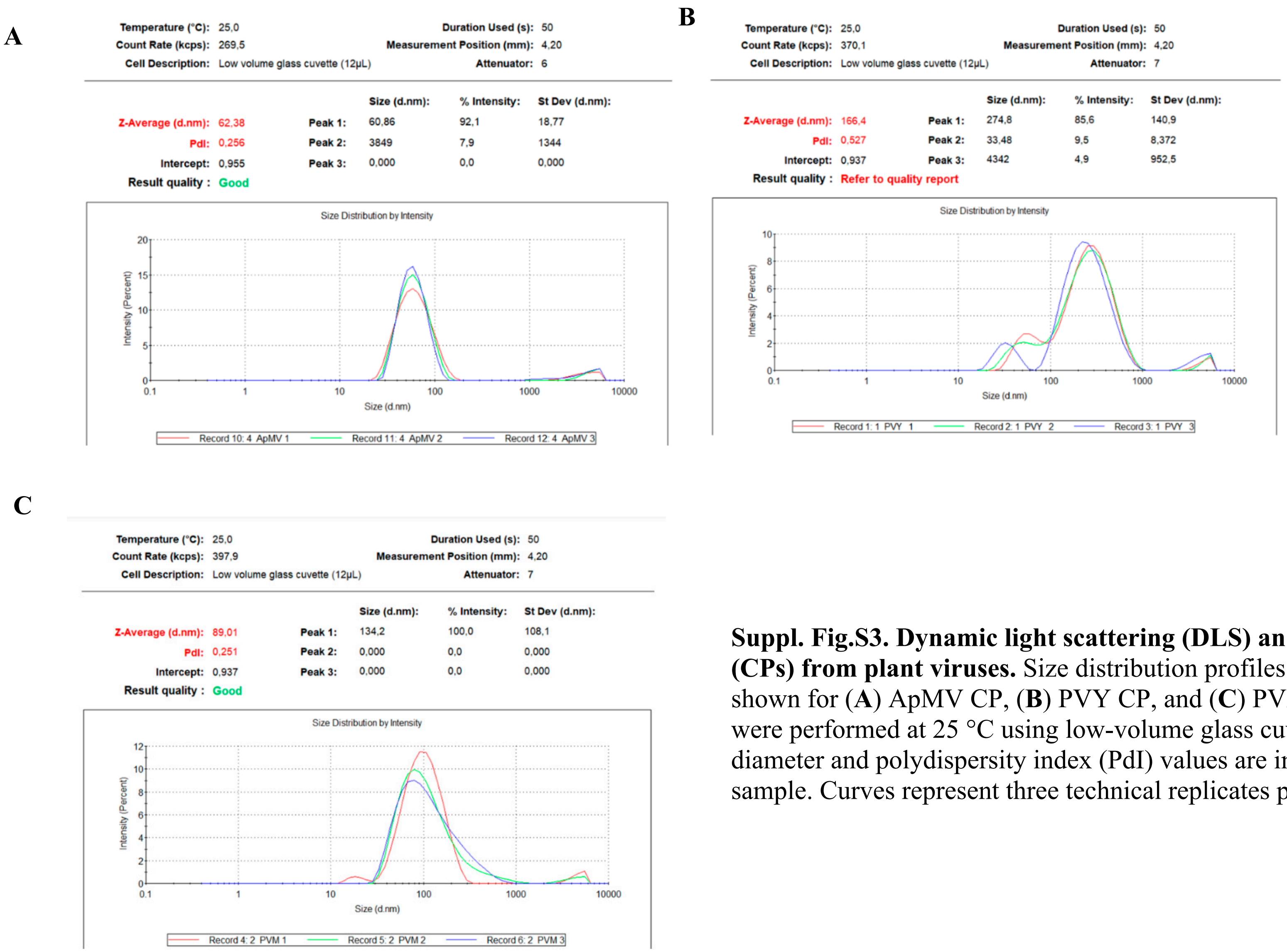

Suppl. Fig.S3. Dynamic light scattering (DLS) analysis of coat proteins (CPs) from plant viruses. Size distribution profiles (intensity-weighted) are shown for (**A**) ApMV CP, (**B**) PVY CP, and (**C**) PVM CP. Measurements were performed at 25 °C using low-volume glass cuvettes. Z-average diameter and polydispersity index (PdI) values are indicated for each sample. Curves represent three technical replicates per protein preparation.

Supplementary Figure S4

A

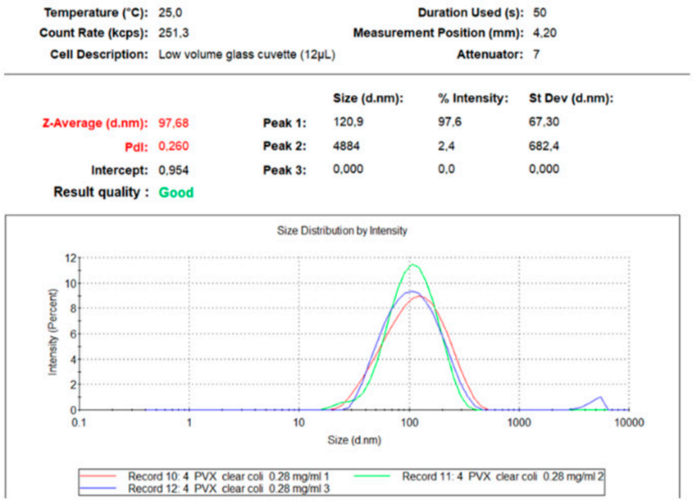

B

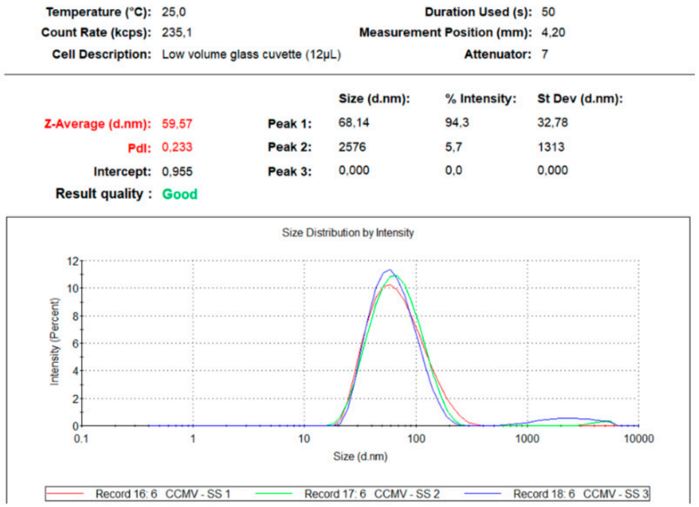

C

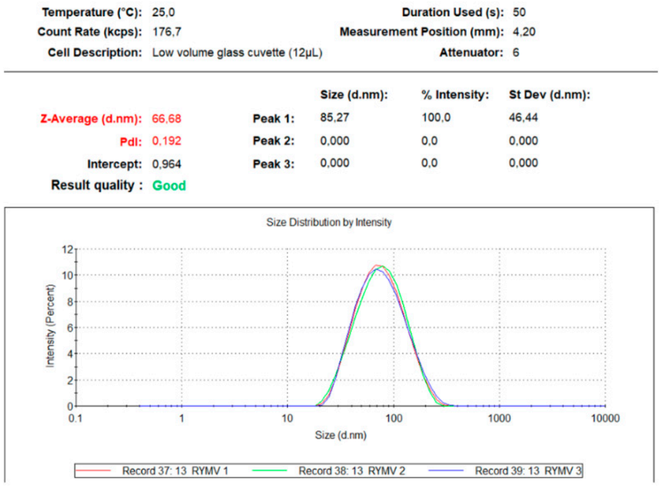

Suppl. Fig.S4. Dynamic light scattering (DLS) analysis of coat proteins (CPs) from plant viruses. Size distribution profiles (intensity-weighted) are shown for (**A**) PVX CP, (**B**) CCMV-ss CP, and (**C**) RYMV CP. Measurements were performed at 25 °C using low-volume glass cuvettes. *Z*-average diameter and polydispersity index (PdI) values are indicated for each sample. Curves represent three technical replicates per protein preparation.

Supplementary Figure S5

A

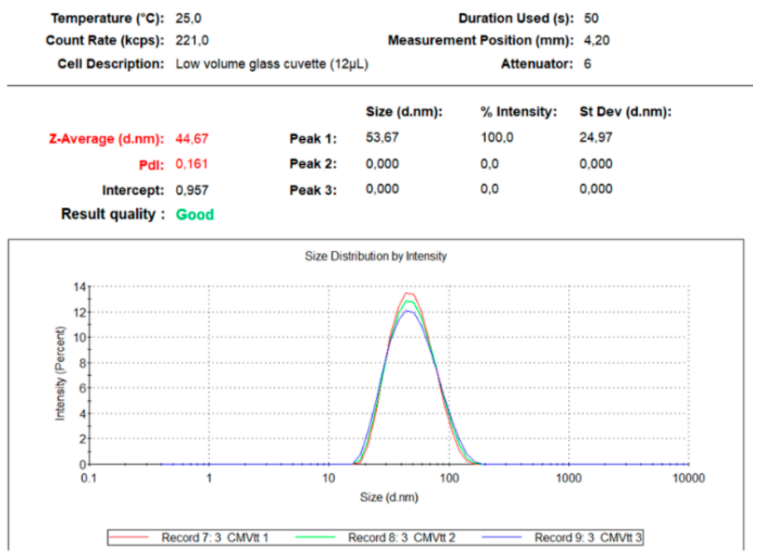

B

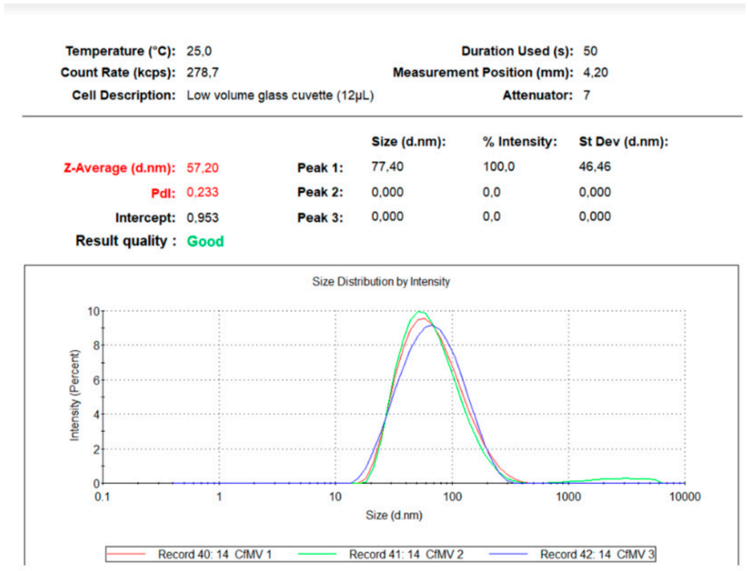

Suppl. Fig.5S. Dynamic light scattering (DLS) analysis of coat proteins (CPs) from plant viruses. Size distribution profiles (intensity-weighted) are shown for (**A**) CMVtt CP and (**B**) CfMV CP. Measurements were performed at 25 °C using low-volume glass cuvettes. Z-average diameter and polydispersity index (PdI) values are indicated for each sample. Curves represent three technical replicates per protein preparation.

Supplementary Figure S6

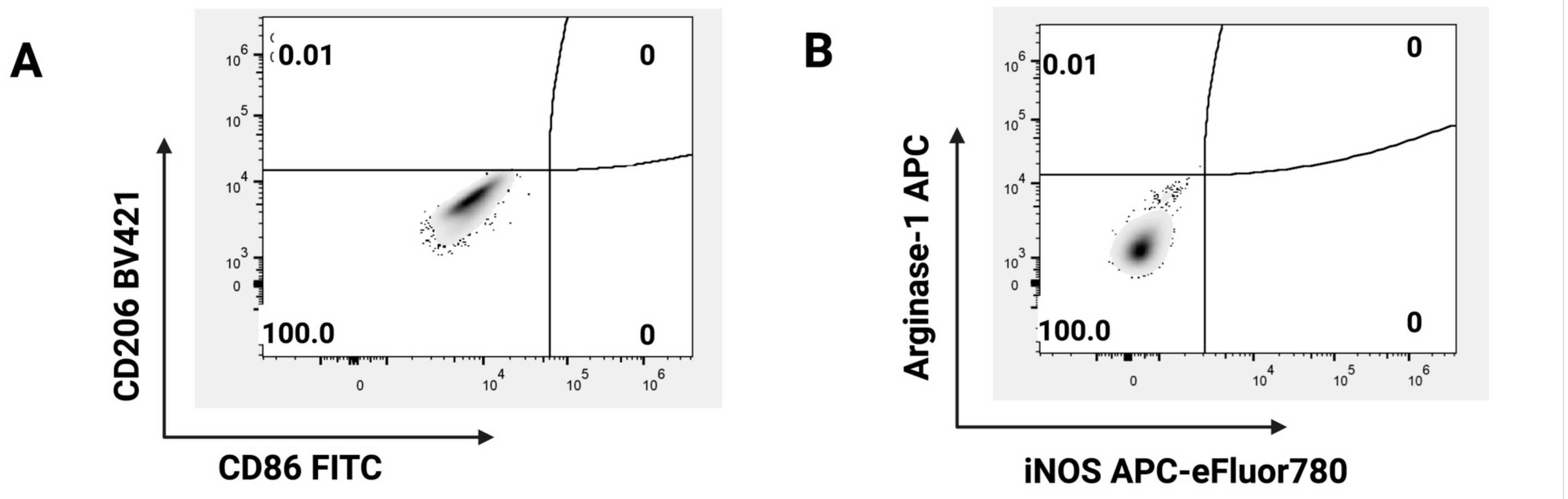

Suppl. Fig. S6. Representative unstained RAW264.7 macrophage controls used to assess background fluorescence in flow-cytometry analysis. Representative unstained RAW264.7 macrophage plots are shown for the CD206/CD86 and Arginase-1/iNOS marker channels. The unstained controls demonstrate background fluorescence in the relevant detection channels and were used to support interpretation of positive staining populations in the macrophage activation assays. Final gate placement was performed using unstained controls together with the corresponding stained samples and reference stimulation controls.

Supplementary Figure S7

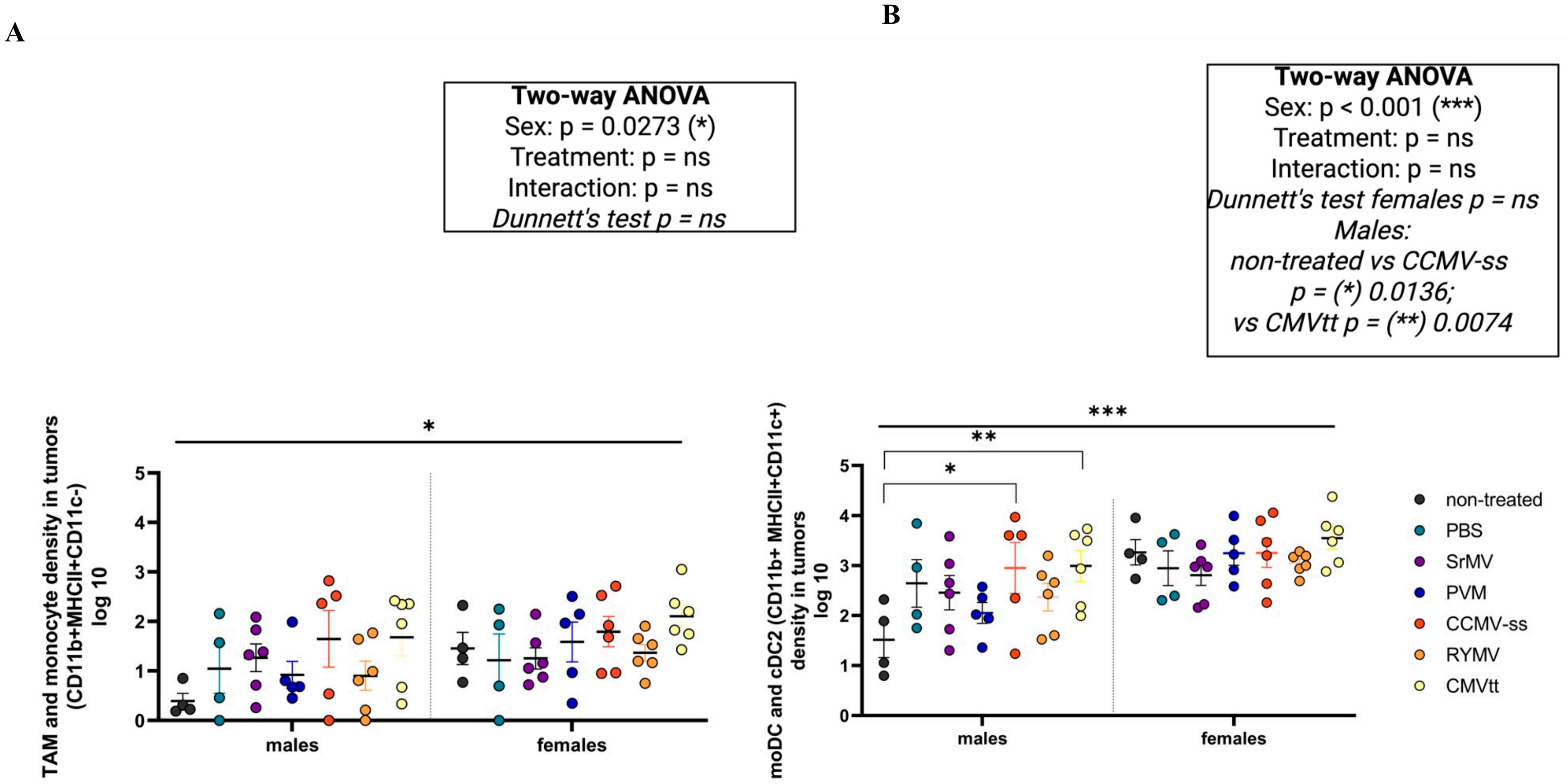

Suppl. Fig. S7. Quantification of tumor-infiltrating myeloid cell populations in excised tumors from male and female mice. Densities (cells per mg tumor tissue) of (**A**) tumor-associated macrophages (TAMs) and monocytes ($CD11b^{+}MHCII^{+}CD11c^{-}$) and (**B**) monocyte-derived dendritic cells (moDC) and conventional dendritic cells type 2 (cDC2; $CD11b^{+}MHCII^{+}CD11c^{+}$) are shown as log_{10}-transformed values. Each dot represents an individual tumor, with horizontal bars indicating group means ± SEM. Statistical analysis was performed using two-way ANOVA to assess effects of host-sex, treatment, and interaction, followed by Dunnett’s multiple-comparison test where indicated. Significance levels are denoted as *$p < 0.05$, **$p < 0.01$.

Supplementary Table S1

Control conditions	Experiment 1 mean RLU	Experiment 2 mean RLU	Experiment 3 mean RLU
Untreated B16-F10-luc2	3425.5	2917.8	2743.3
B16-F10-luc2 + SrMV VLPs	3395.3	2161.5	2407.3
B16-F10-luc2 + PVM VLPs	3152.5	2208.3	2370.5
B16-F10-luc2 + CCMV-ss VLPs	3305.1	2428.1	2784.7
B16-F10-luc2 + RYMV VLPs	3059.2	2364.6	1780.1
B16-F10-luc2 + CMVtt VLPs	2346.6	1285.8	808.3
B16-F10-luc2 + LPS/IFN-γ	1862.0	1550.3	1400.4
B16-F10-luc2 + IL-4	1874.4	1263.5	1356.9

Suppl. Table S1. Condition-matched B16-F10-luc2 stimulus-only controls used for co-culture normalization. Values represent mean raw luminescence signals from three independent experiments and are shown separately for each experiment because absolute luminescence values differed between experiments. These B16-F10-luc2 stimulus-only controls were used as condition-matched tumor-cell survival references for normalization of the corresponding co-culture conditions and were not analyzed as an independent cytotoxicity endpoint. RLU – relative light units.
